# Genetic and Inflammatory Signatures Associated With Worse Prognosis in Hospitalized Patients With Severe SARS‐CoV‐2 Infection With and Without Diabetes

**DOI:** 10.1002/jmv.70425

**Published:** 2025-06-06

**Authors:** Marshall Yuan, Andrew Wassef, Davit Sargsyan, Vahe Nersisyan, Javier Cabrera, Ronald G. Nahass, Wael Hassan, Ah‐Ng Kong, Luigi Brunetti

**Affiliations:** ^1^ Robert Wood Johnson Medical School Piscataway New Jersey USA; ^2^ Department of Pharmaceutics Ernest Mario School of Pharmacy Piscataway New Jersey USA; ^3^ Johnson & Johnson, Translational Medicine and Early Development Statistics Raritan New Jersey USA; ^4^ Department of Statistics Rutgers University Piscataway New Jersey USA; ^5^ IDCare Hillsborough New Jersey USA; ^6^ Robert Wood Johnson University Hospital Somerset Somerville New Jersey USA; ^7^ Department of Pharmacy Practice Ernest Mario School of Pharmacy Piscataway New Jersey USA

**Keywords:** cytokine/chemokine, gene expression, SARS coronavirus

## Abstract

Severe acute respiratory syndrome coronavirus 2 (SAR‐CoV‐2) presents with a diverse symptomology, ranging from asymptomatic to severe disease, but the mechanism of risk factors such as diabetes remains unelucidated. The current retrospective cohort study of 182 patients, with and without COVID‐19 and diabetes, analyzed leftover blood specimens for RNA sequencing and chemokine/cytokine, ACE2/DPP‐IV concentrations. After analysis, 14 223 genes had sufficient hits; 18 genes and 431 genes were differentially expressed between patients with and without COVID‐19 and patients with and without diabetes, respectively. Both analyses had differentially expressed five genes, GRASP, KRT8, MYZAP, PRKG1, and SMIM24. DPP‐IV concentrations were statistically lower in COVID‐19 patients versus non‐COVID‐19, but no significant differences in chemokine/cytokine expression and ACE2 concentrations were detected. This study provides insight into altered gene expression patterns in individuals with COVID‐19 with and without diabetes mellitus and highlights potential markers for severe disease and pathways for treatment targets.

## 1 Introduction

Coronavirus Disease of 2019 (COVID‐19), caused by the Severe Acute Respiratory Syndrome Coronavirus 2 (SARS‐CoV‐2), was announced as a pandemic by the WHO at the beginning of 2020 due to its rapid communicability and disease severity [1]. Although the genesis of disease spread is not entirely understood, the interplay between COVID‐19's bat zoonotic origins and climate change may have contributed to the initial human exposure and eventual propagation [2, 3]. The primary route of SARS‐CoV‐2 transmission occurs through aerosolized particles emitted from the respiratory tract of an infected person to a contact [4, 5]. After exposure, an innate immune response is followed by antigen‐specific adaptive immunity, which is mediated by B cells (humoral immunity) that produce neutralizing antibodies, and T cells (cellular immunity) [6].

Primarily a condition that affects the respiratory system, the disease presents in patients with a wide range of symptoms, ranging from asymptomatic and mild to severe. Fortunately, advancements in the prevention of COVID‐19 have revolutionized the approach to the disease. Both mRNA and nanovaccines have been utilized to deliver mRNA that codes for the SARS‐CoV‐2 spike protein, which produces a highly effective immune response capable of providing protection against COVID‐19 [7, 8]. In the most critical cases of infection, however, patients may require intensive critical care (ICU) and mechanical intubation, among other intensive interventions [1]. Several antivirals are available for the treatment of infection, such as nirmatrelvir with ritonavir, remdesivir, and molnupiravir. Numerous other therapies have been suggested, such as vitamin D, convalescent plasma, colchicine, and hydroxychloroquine, but data are insufficient to support their use [9, 10, 11, 12, 13]. Additionally, new technologies are under consideration, including microRNA, nanoparticles, and novel antivirals [14, 15, 16]. A variety of risk factors are suggested to increase the risk for severe illness, including age greater than 65 years, hypertension, smoking, and diabetes [17].

Multiple meta‐analyses of the clinical correlation between diabetes and SARS‐CoV‐2 have demonstrated that individuals with diabetes are at higher risk for severe disease and mortality, reporting odds ratios as high as OR = 2.75 (95% CI: 2.09–3.62; *p* < 0.01) for severe disease [1, 18, 19]. Diabetes has been previously implicated in other infectious conditions, including being associated with over a fourfold risk of ICU admission in patients with the Influenza A infection of 2009 (H1N1) [20]. Furthermore, diabetes has been observed to be associated with critical illness and identified as an independent risk factor for 90‐day mortality in patients with Middle East respiratory syndrome coronavirus (MERS‐CoV) [21]. Other studies further corroborate a bidirectional link between diabetes and COVID‐19, including cases and systematic reviews that found a higher incidence rate of new‐onset diabetes and hyperglycemia in patients previously infected by COVID‐19 [22, 23]. Despite the substantial data that supports diabetes as a risk factor for COVID‐19, the mechanism that mediates this risk is largely unknown.

Although poorly elucidated, the mechanism of disease severity in diabetes mellitus patients may be connected to angiotensin‐converting enzyme 2 (ACE2) and cytokine/chemokine gene expression [24]. Severe acute respiratory syndrome coronavirus‐2 (SARS‐CoV‐2) uses the ACE2 receptor to enter host cells [25]. Upon entry, there is a downregulation of surface ACE2 expression. Circulating angiotensin 2 (Ang‐II) is elevated in COVID‐19 patients compared to healthy controls, providing evidence of renin‐angiotensin system (RAS) imbalance in the disease [26]. Increases in Ang‐II lead to increases in disintegrin and metalloproteinase 17 (ADAM17) activity and subsequent release of tumor necrosis factor α (TNF‐α) and other inflammatory cytokines [25]. Nuclear factor erythroid 2–related factor 2 (NRF2) and NRF2‐related genes regulate cellular redox balance and release inflammatory cytokines and chemokines secondary to stress. NRF2 activation downregulated a variety of cytokines that were reported to be elevated in COVID‐19, suggesting reduced NRF2 activity as a contributor to the “cytokine storm” seen in COVID‐19 [27].

Chemokines are an important secretory protein responsible for immune signaling and have been implicated in various lung pathologies. For example, CCL2 [chemokine (C‐C motif) ligand 2; monocyte chemoattractant protein‐1, (MCP‐1)] and its receptor CCR2 are involved in monocyte/macrophage migration, Th2 cell polarization, and the production of TGF‐β and procollagen in fibroblast cells [28, 29]. This chemokine is associated with acute respiratory distress syndrome and pulmonary fibrosis [30]—both observed in COVID‐19. CCL2 elevation has also been found to be associated with severe SARS‐CoV [31]. Various chemokines have been reported to be elevated in COVID‐19 infection, but differential expression patterns have not been evaluated in individuals with and without diabetes [32]. This study assessed gene and protein expression patterns in individuals hospitalized with diabetes mellitus infected with SARS‐CoV‐2 and examined the relationship between these patterns and disease severity.

## 2 Methods

### 2.1 Data Source and Sample Collection

We performed a single‐center, IRB‐approved cohort study (Robert Wood Johnson University Hospital Institutional Review Board; IRB20‐16) using data from electronic health records and leftover clinical specimens at a large community medical center. All subjects 18 years of age or older with remnant clinical blood specimens within 48 h of hospital admission were eligible for inclusion. Pregnant patients and those discharged directly from the Emergency Department were excluded. An aliquot of leftover whole blood specimens collected in EDTA tubes was immediately frozen for each patient. The remaining whole blood was centrifuged at 3000*g* for 10 min, and plasma was drawn off. All samples were stored at −80°C until analysis.

### 2.2 Data Extraction and Collection

All data were extracted from the electronic health record (EPIC Systems; Wisconsin, USA). The records included patient age, sex, race/ethnicity, comorbidities, vaccination status, concomitant medications, COVID‐related treatment interventions, and other relevant clinical laboratory data. Patient comorbidities were identified using the International Classification of Diseases, tenth revision, clinical modification (ICD‐10‐CM) codes. The overall comorbidity status of patients was defined by the scoring of the Charlson–Deyo Comorbidity Index (CCI).

### 2.3 RNA‐Sequencing

Singulomics (Bronx, NY) performed RNA sequencing “fee‐for‐service”. RNA was purified using poly‐T oligo‐attached magnetic beads. After fragmentation, the first strand of cDNA was synthesized using random hexamer primers. This was followed by synthesizing the second strand of cDNA using either dUTP for a directional library or dTTP for a nondirectional library. The nondirectional library was ready after end repair, A‐tailing, adapter ligation, size selection, amplification, and purification.

### 2.4 Cytokine and Chemokine Multiplex Assay

Plasma cytokine and chemokine concentrations were measured using ProcartaPlex Human Cytokine Storm 21‐Plex (Invitrogen; EPX210‐15850‐901) on the Luminex platform. The multiplex panel measured the plasma concentration of IFN‐α, IFN‐γ, IL‐1β, IL‐2, IL‐4, IL‐5, IL‐6, IL‐8 (CXCL8), IL10, IL‐12p70, IL‐17A (CTLA‐8), IL‐18, IP‐10 (CXCL10), MCP‐1 (CCL2), MIP‐1α, MIP‐1β, TNF‐α, and TNFβ. Briefly, 25 µL of plasma and internal controls were plated on a 96‐well plate, incubated with magnetic beads, and washed before adding 25 μL of detection antibody. The plate was then incubated for 30 min, followed by adding 50 μL of Streptavidin‐PE to each well. The concentration of analytes was reported as pg/mL.

### 2.5 ACE2 and DPP‐IV ELISA

Circulating ACE2 and DPP‐IV were measured by sandwich ELISA (Invitrogen; EH489RB; Invitrogen; EHDPP4). Briefly, 100 μL of standards and 100 μL of diluted plasma samples using the assay‐specific diluent were plated on a 96‐well plate. After a series of washes, 100 μL of biotin was added to each well, followed by a 1‐h incubation period at room temperature with gentle shaking. The solution was discarded, the plate was washed, and 100 μL of streptavidin‐HRP was added. The plate was incubated for 45 min with gentle shaking. After the solution was discarded and the plate washed, 100 μL of TMB substrate was added and incubated for 30 min. Once the stop solution was added, the plate was read at an absorbance of 450 nm, and an assay‐specific standard curve was used to obtain the protein concentrations. The plasma ACE2 and DPP‐IV concentrations were reported as ng/mL and pg/mL, respectively.

### 2.6 Primary and Secondary Outcomes

Patients were stratified into those with and without COVID‐19, as well as those with and without diabetes. The primary endpoint was the identification of differentially expressed genes between individuals with and without COVID‐19 stratified by diabetes status. Secondary endpoints included differences in inflammatory mediator expression, circulating ACE2, and circulating DPPIV, as well as clinical outcomes such as death, length of hospital stay, and WHO‐OSCI score.

### 2.7 Statistical Analysis

Statistical analysis and data visualization were performed using R 4.3.1 software [33]. All data are presented with summary statistics. Categorical variables are represented as proportions, and continuous data are represented by means and standard deviations or standard errors. Differences in baseline characteristics were analyzed using a two‐sample *t*‐test or an analysis of variance (ANOVA) for more than two samples of continuous data, and a *χ* ^2^ test for categorical data. RNA‐seq data were analyzed with the DESeq. 2R package based on the negative binomial distribution for differential gene expression analysis [34].

ELISA data was analyzed using a two‐part linear model that fits a logistic model to the binary portion of the data (zero or non‐zero) and a linear model to the non‐zero part [35, 36, 37]. The average marginal effects of the two‐part model estimates and the 95% confidence intervals (CI) for the estimates were visualized to present the results.

## 3 Results

**Figure 1:**
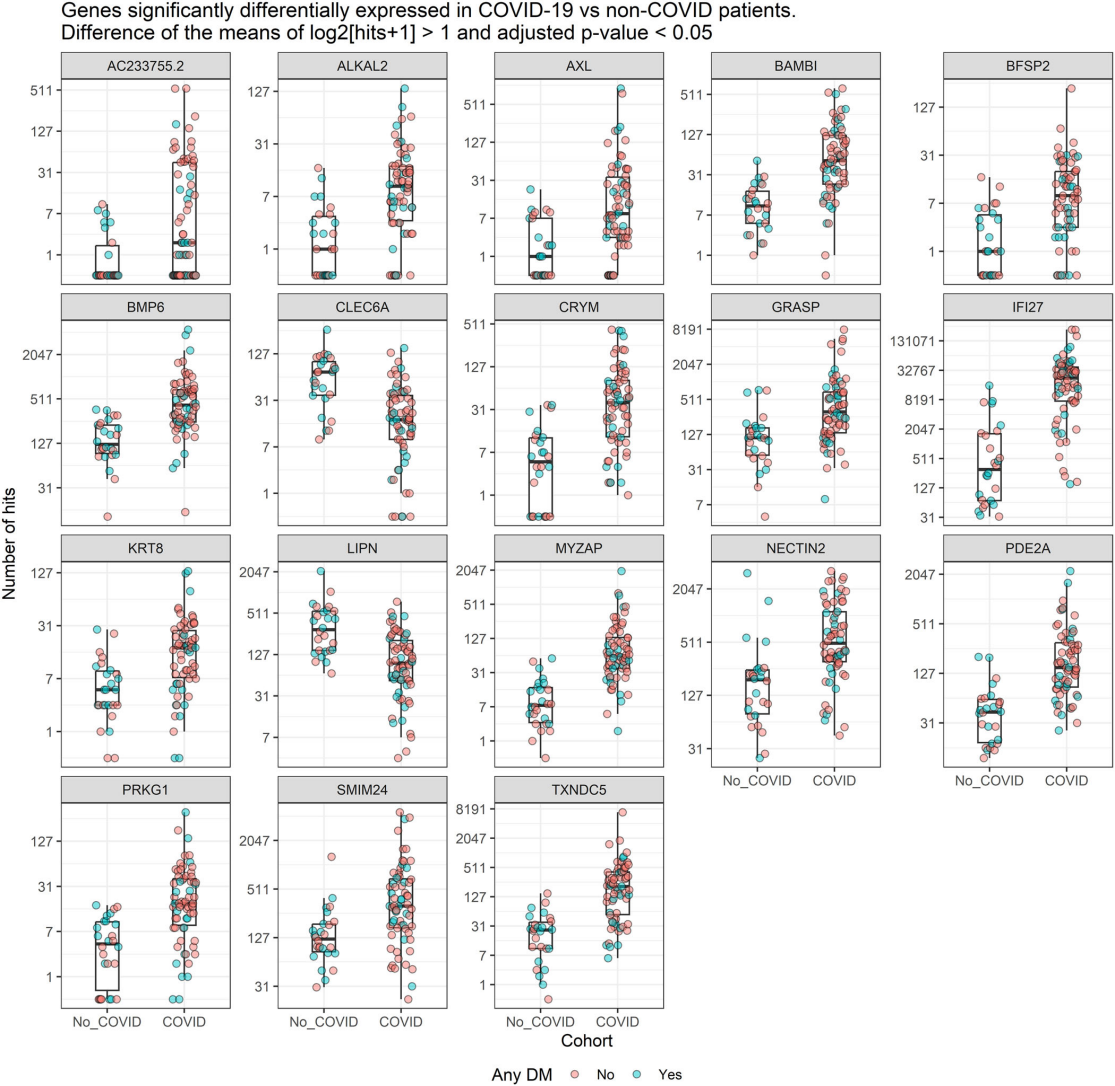
Genes significantly differentially expressed in COVID‐19 vs. non‐COVI‐19 patients. Difference of the means of log2[hits+1] > 1 and adjusted *p* value < 0.05.

The study included 182 hospitalized adult patients with an admitting diagnosis of COVID‐19 (*n* = 110) and control subjects admitted for any other reason (*n *= 72). All available remnant blood samples were obtained within 48 h of hospital presentation. Table 1 summarizes patients' baseline characteristics, including demographics and comorbidities, stratified by COVID‐19 and diabetes mellitus (DM) diagnosis. Graphical representation of baseline characteristics is shown in Supporting Information S1: Figure 1. Overall, individuals with DM, regardless of COVID‐19 status, had higher comorbidity, as described by the Charlson Comorbidity Index (CCI). Available clinical laboratory values are summarized in Supporting Information S5: Table 1. A significant proportion of laboratory values were missing for the no COVID‐19 group, likely due to the patient's diagnosis and the necessity for a specific laboratory order. Individuals with COVID‐19 were more likely to be on remdesivir, tocilizumab, and corticosteroids (Supporting Information S5: Table 2). In terms of background diabetes therapeutics, all individuals with DM, regardless of COVID‐19 status, received similar medications. Individuals with DM were more likely to receive anticoagulants, and those with COVID‐19 were more likely to be on full‐dose anticoagulation (81.8% vs*.* 72.2%).

**Table 1:** Subject demographic and clinical characteristics stratified by the presence and absence of diabetes and COVID‐19.

	No COVID		COVID		Overall		*p* value*
	No DM (*N* = 39)	Any DM (*N* = 33)	No DM (*N* = 76)	Any DM (*N* = 34)	No DM (*N* = 115)	Any DM (*N* = 67)	
Hospital disposition							0.993
Inpatient	34 (87.2%)	30 (90.9%)	69 (90.8%)	31 (91.2%)	103 (89.6%)	61 (91.0%)	
ER	4 (10.3%)	2 (6.1%)	5 (6.6%)	2 (5.9%)	9 (7.8%)	4 (6.0%)	
OP	1 (2.6%)	1 (3.0%)	2 (2.6%)	1 (2.9%)	3 (2.6%)	2 (3.0%)	
Age							0.388
Mean (SD)	67.5 (18.1)	64.2 (16.8)	61.4 (16.4)	67.0 (13.7)	63.5 (17.2)	65.6 (15.2)	
Median [Min, Max]	74.0 [22.0, 92.0]	66.0 [28.0, 94.0]	61.5 [21.0, 93.0]	68.5 [36.0, 89.0]	64.0 [21.0, 93.0]	68.0 [28.0, 94.0]	
Sex							0.544
Male	18 (46.2%)	17 (51.5%)	45 (59.2%)	20 (58.8%)	63 (54.8%)	37 (55.2%)	
Female	21 (53.8%)	16 (48.5%)	31 (40.8%)	14 (41.2%)	52 (45.2%)	30 (44.8%)	
Race							0.005
White non‐Hispanic	27 (69.2%)	18 (54.5%)	48 (63.2%)	24 (70.6%)	75 (65.2%)	42 (62.7%)	
Black	3 (7.7%)	3 (9.1%)	7 (9.2%)	1 (2.9%)	10 (8.7%)	4 (6.0%)	
Asian	4 (10.3%)	2 (6.1%)	14 (18.4%)	6 (17.6%)	18 (15.7%)	8 (11.9%)	
Hispanic	3 (7.7%)	3 (9.1%)	7 (9.2%)	3 (8.8%)	10 (8.7%)	6 (9.0%)	
Other	2 (5.1%)	7 (21.2%)	0 (0%)	0 (0%)	2 (1.7%)	7 (10.4%)	
Weight (kg)							0.407
Mean (SD)	80.5 (24.5)	85.7 (22.6)	90.6 (30.8)	88.8 (22.0)	87.2 (29.1)	87.3 (22.2)	
Median [Min, Max]	73.1 [48.0, 134]	89.4 [45.4, 141]	86.0 [42.0, 217]	84.8 [45.2, 154]	82.3 [42.0, 217]	84.9 [45.2, 154]	
BMI							0.368
Mean (SD)	27.8 (6.97)	29.5 (6.49)	31.5 (9.87)	31.9 (7.35)	30.3 (9.13)	30.7 (6.99)	
Median [Min, Max]	25.8 [18.9, 47.7]	29.1 [16.7, 43.9]	29.0 [20.0, 71.6]	31.6 [19.5, 50.7]	28.0 [18.9, 71.6]	29.8 [16.7, 50.7]	
Obesity	16 (41.0%)	15 (45.5%)	33 (43.4%)	19 (55.9%)	49 (42.6%)	34 (50.7%)	0.588
Obesity Class							0.629
I	11 (28.2%)	8 (24.2%)	13 (17.1%)	11 (32.4%)	24 (20.9%)	19 (28.4%)	
II	3 (7.7%)	4 (12.1%)	10 (13.2%)	5 (14.7%)	13 (11.3%)	9 (13.4%)	
III	2 (5.1%)	2 (6.1%)	9 (11.8%)	3 (8.8%)	11 (9.6%)	5 (7.5%)	
Charlson Comorbidity Index							0.096
Mean (SD)	5.26 (3.82)	6.39 (3.79)	2.22 (1.87)	4.24 (2.43)	3.25 (3.04)	5.30 (3.33)	
Median [Min, Max]	5.00 [0, 14.0]	6.00 [1.00, 18.0]	2.00 [0, 7.00]	3.50 [1.00, 9.00]	3.00 [0, 14.0]	5.00 [1.00, 18.0]	
Myocardial infarction	6 (15.4%)	5 (15.2%)	0 (0%)	1 (2.9%)	6 (5.2%)	6 (9.0%)	0.002
Heart failure	12 (30.8%)	6 (18.2%)	1 (1.3%)	5 (14.7%)	13 (11.3%)	11 (16.4%)	< 0.001
Peripheral vascular disease	2 (5.1%)	2 (6.1%)	1 (1.3%)	1 (2.9%)	3 (2.6%)	3 (4.5%)	0.542
Cerebrovascular disease	9 (23.1%)	10 (30.3%)	2 (2.6%)	1 (2.9%)	11 (9.6%)	11 (16.4%)	< 0.001
Dementia	2 (5.1%)	2 (6.1%)	6 (7.9%)	5 (14.7%)	8 (7.0%)	7 (10.4%)	0.458
COPD	5 (12.8%)	6 (18.2%)	9 (11.8%)	5 (14.7%)	14 (12.2%)	11 (16.4%)	0.841
Rheum/connective tissue disease	2 (5.1%)	1 (3.0%)	1 (1.3%)	0 (0%)	3 (2.6%)	1 (1.5%)	0.440
Peptic ulcer disease	2 (5.1%)	1 (3.0%)	0 (0%)	0 (0%)	2 (1.7%)	1 (1.5%)	0.161
Mild liver disease	2 (5.1%)	0 (0%)	0 (0%)	2 (5.9%)	2 (1.7%)	2 (3.0%)	0.104
Hemoplegia	1 (2.6%)	3 (9.1%)	0 (0%)	0 (0%)	1 (0.9%)	3 (4.5%)	0.020
Renal disease	5 (12.8%)	11 (33.3%)	4 (5.3%)	5 (14.7%)	9 (7.8%)	16 (23.9%)	0.002
Cancer	5 (12.8%)	4 (12.1%)	0 (0%)	0 (0%)	5 (4.3%)	4 (6.0%)	< 0.001
Moderate‐to‐severe liver	2 (5.1%)	1 (3.0%)	0 (0%)	0 (0%)	2 (1.7%)	1 (1.5%)	0.161
Metastatic cancer**	6 (15.4%)	5 (15.2%)	0 (0%)	0 (0%)	6 (5.2%)	5 (7.5%)	< 0.001
AIDS	0 (0%)	1 (3.0%)	0 (0%)	0 (0%)	0 (0%)	1 (1.5%)	0.300
Hypertension	26 (66.7%)	26 (78.8%)	39 (51.3%)	30 (88.2%)	65 (56.5%)	56 (83.6%)	0.001
Hyperlipidemia	24 (61.5%)	23 (69.7%)	23 (30.3%)	26 (76.5%)	47 (40.9%)	49 (73.1%)	< 0.001
Metabolic syndrome criteria							< 0.001
0	11 (28.2%)	0 (0%)	22 (28.9%)	0 (0%)	33 (28.7%)	0 (0%)	
1	6 (15.4%)	3 (9.1%)	19 (25.0%)	2 (5.9%)	25 (21.7%)	5 (7.5%)	
2	22 (56.4%)	10 (30.3%)	23 (30.3%)	3 (8.8%)	45 (39.1%)	13 (19.4%)	
3	0 (0%)	19 (57.6%)	9 (11.8%)	15 (44.1%)	9 (7.8%)	34 (50.7%)	
4	0 (0%)	1 (3.0%)	3 (3.9%)	14 (41.2%)	3 (2.6%)	15 (22.4%)	

* *p* values are from analysis of variance (ANOVA) tests for continuous data and *χ* ^2^ test for categorical data, with each condition tested across four groups: No COVID/no DM, No COVID/Any DM, COVID/No DM, and COVID/Any DM.

** Solid cancers only.

### 3.1 Differential Gene Expression Analyses

In total, 58 708 protein‐coding and long noncoding genes and gene variants were found in the 92 RNA‐seq samples. Of these, 19 909 were protein‐coding genes. Note that 15 genes had two variants each. For these genes, the variant counts were added up within each sample.

**Figure 2:**
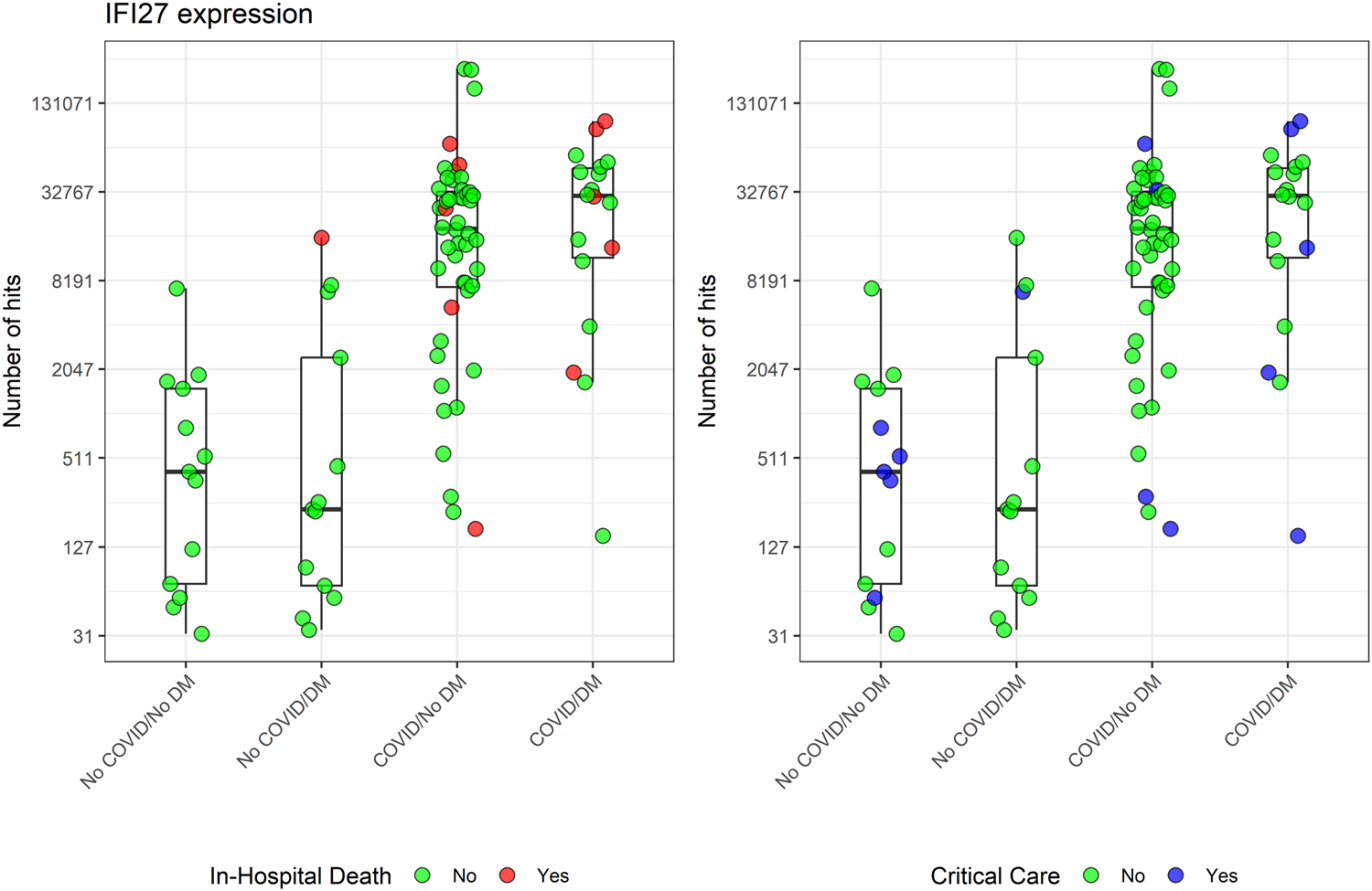
IFI27 expression by COVID and DM, color‐coded for in‐hospital deaths (left) and critical care (right).

Genes with a small number of hits were filtered out. After examining the number of genes remaining after filtering using varying minimum numbers of hits per sample and the minimum number of samples with at least that many hits (Supporting Information S2: Figure 2), it was decided to set both numbers to 10. Hence, 14 223 genes with at least ten hits in at least 10 out of 92 samples were used in the analysis.

Next, we identified coding genes differentially expressed in COVID patients compared to controls (at least twofold change and false discovery rate (FDR) adjusted *p*value ≤ 0.05). Two genes were downregulated and 16 upregulated in COVID patients compared to non‐COVID. The list of the 18 genes and the estimates of the differences (on a log2 scale and representing the number of twofold changes in COVID vs. non‐COVID patients' samples) is presented in Table 2. Figure 1 shows the total number of hits for each of the 18 genes in each sample.

**Table 2:** Differentially expressed genes in hospitalized patients with COVID‐19 vs. hospitalized patients admitted for other acute illnesses.

Gene	Median hits in non‐COVID	Median hits in COVID	Log2 fold change (SE)	Adjusted *p* value
AC233755.2	0	2	3.43 (0.79)	0.016
ALKAL2	1	9.5	1.91 (0.46)	0.028
AXL	1	8.5	2.36 (0.52)	0.015
BAMBI	10	51	1.42 (0.33)	0.016
BFSP2	1	9	1.81 (0.41)	0.016
BMP6	124	421	1.10 (0.25)	0.016
CLEC6A	73.5	17	‐1.46 (0.35)	0.028
CRYM	5	39	1.82 (0.43)	0.024
GRASP	112	312	1.57 (0.33)	0.010
IFI27	310	22 806	2.37 (0.49)	0.007
KRT8	5	17	1.24 (0.31)	0.040
LIPN	294	95.5	−1.36 (0.32)	0.019
MYZAP	7.5	62.5	1.42 (0.35)	0.040
NECTIN2	191	499	1.18 (0.28)	0.024
PDE2A	42.5	149.5	1.41 (0.29)	0.007
PRKG1	4.5	18	1.53 (0.38)	0.041
SMIM24	122	315	1.58 (0.39)	0.040
TXNDC5	25.5	207.5	1.63 (0.37)	0.016

One of the most striking differences found in this part of the analysis was the overexpression of the Interferon‐Alpha Inducible Protein 27 (IFI27) coding gene, with more than a twofold difference (Ratio = 2.37, SEM= 0.49) in patients with COVID versus non‐COVID. In this study, most patients who died in the hospital had elevated IFI27 expression levels compared to those discharged alive (Figure 2, left panel). No apparent patterns were observed for individuals requiring more intensive care while hospitalized (Figure 2, right panel).

**Figure 3:**
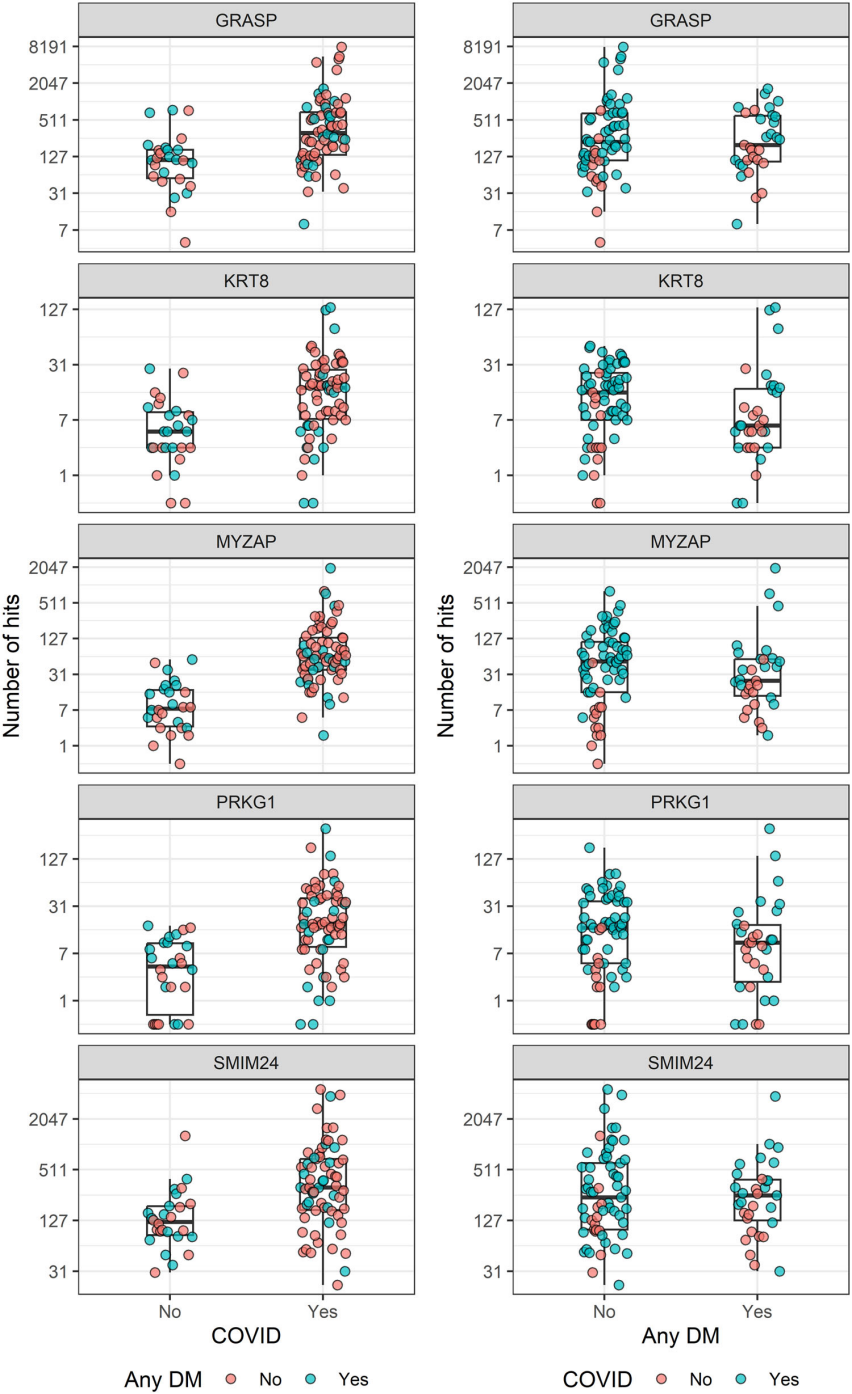
Number of hits for specific differentially expressed genes in samples, grouped by COVID and DM diagnoses.

Out of all genes found to be differentially expressed in COVID/non‐COVID and DM/non‐DM patients, there were five genes in common: GRASP, KRT8, MYZAP, PRKG1, and SMIM24. The number of hits in the samples, grouped by COVID and DM diagnoses, are presented in Figure 3.

### 3.2 Inflammatory Signature Analysis

**Figure 4:**
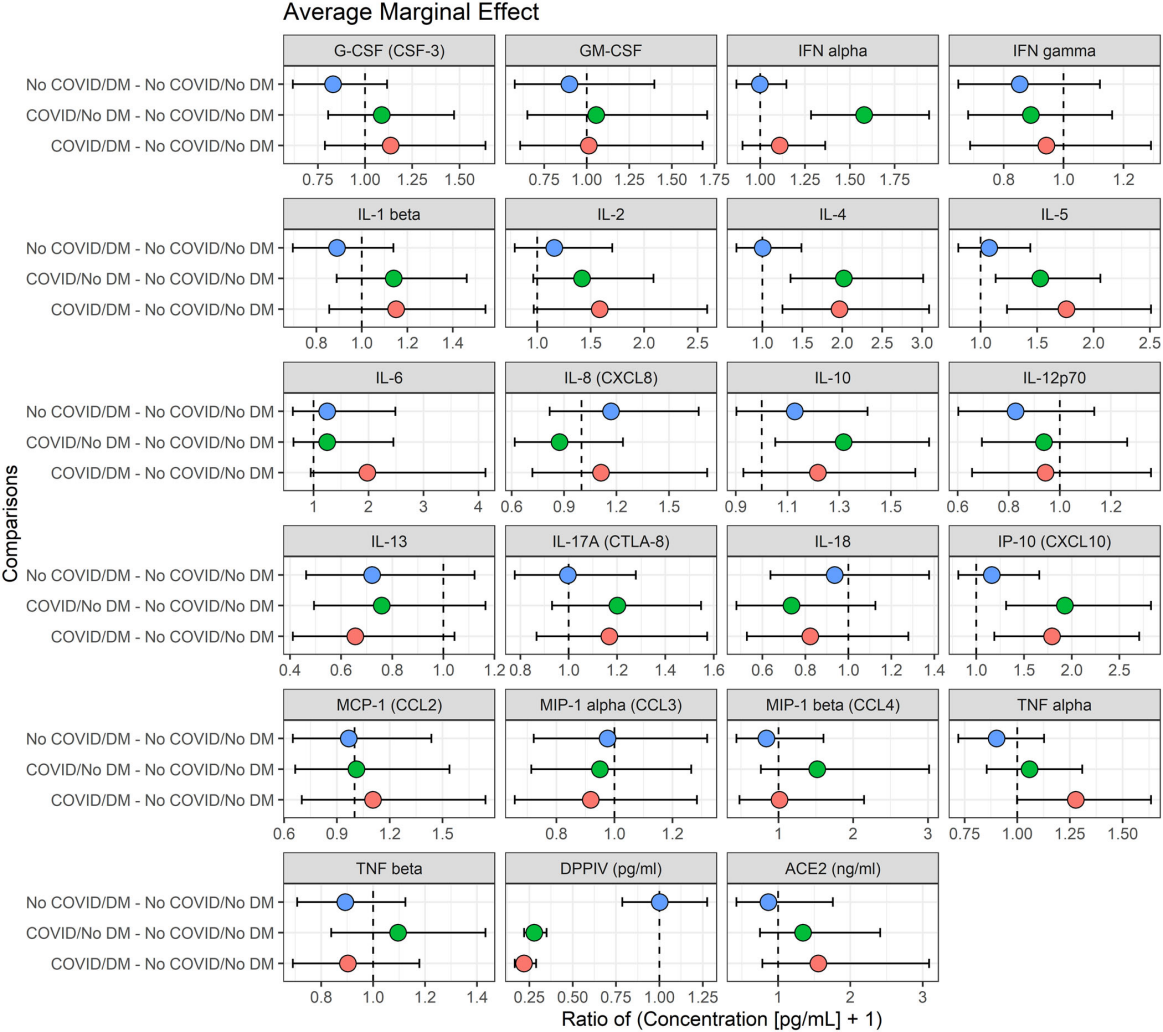
Comparison of select cytokine and chemokine differences between individuals with and without COVID‐19 and diabetes mellitus (DM).

Cytokines and chemokines were measured using a multiplex ELISA assay. In total, 21 plasma protein concentrations were measured in 54 COVID and 68 non‐COVID patients (Table 3, Figure 4, and Supporting Information S3: Figure 3). IFN alpha and IL‐10 were increased in individuals with COVID‐19 without DM versus those without either disease. IL‐4, IL‐5, and IP‐10 in all individuals with COVID versus those without infection, regardless of DM status. There was a numerical increase in TNF alpha and IL‐6 in individuals with COVID and DM versus those without either disease; however, the difference failed to reach significance.

**Table 3:** Plasma inflammatory cytokine and chemokine comparison between subjects with and without COVID‐19 and diabetes mellitus.

	No COVID		COVID		Overall		*p* value
	No DM (*N* = 39)	Any DM (*N* = 33)	No DM (*N* = 76)	Any DM (*N* = 34)	No DM (*N* = 115)	Any DM (*N* = 67)	
G‐CSF (CSF‐3)							0.203
Mean (SD)	1.83 (2.65)	1.10 (1.28)	2.17 (2.85)	1.97 (1.77)	1.99 (2.73)	1.44 (1.53)	
Median [Min, Max]	1.04 [0, 11.6]	0.880 [0, 5.04]	1.19 [0, 10.5]	1.83 [0, 6.11]	1.19 [0, 11.6]	1.04 [0, 6.11]	
Missing	1 (2.6%)	3 (9.1%)	41 (53.9%)	15 (44.1%)	42 (36.5%)	18 (26.9%)	
GM‐CSF							0.196
Mean (SD)	4.38 (15.6)	1.30 (4.26)	2.54 (6.67)	1.12 (2.25)	3.50 (12.1)	1.23 (3.59)	
Median [Min, Max]	0 [0, 77.1]	0 [0, 19.1]	0 [0, 25.7]	0 [0, 6.59]	0 [0, 77.1]	0 [0, 19.1]	
Missing	1 (2.6%)	3 (9.1%)	41 (53.9%)	15 (44.1%)	42 (36.5%)	18 (26.9%)	
IFN alpha							0.975
Mean (SD)	0.0754 (0.236)	0.0648 (0.156)	1.28 (2.57)	0.242 (0.565)	0.652 (1.88)	0.133 (0.377)	
Median [Min, Max]	0 [0, 1.30]	0 [0, 0.743]	0.390 [0, 11.7]	0.0650 [0, 2.42]	0 [0, 11.7]	0 [0, 2.42]	
Missing	1 (2.6%)	3 (9.1%)	41 (53.9%)	15 (44.1%)	42 (36.5%)	18 (26.9%)	
IFN gamma							0.068
Mean (SD)	3.12 (3.63)	1.98 (1.92)	1.92 (1.09)	2.41 (2.55)	2.54 (2.77)	2.15 (2.17)	
Median [Min, Max]	1.34 [0.290, 14.4]	1.26 [0.145, 8.51]	1.89 [0, 4.50]	1.49 [0.415, 11.6]	1.71 [0, 14.4]	1.37 [0.145, 11.6]	
Missing	1 (2.6%)	3 (9.1%)	41 (53.9%)	15 (44.1%)	42 (36.5%)	18 (26.9%)	
IL‐1 beta							0.373
Mean (SD)	0.859 (1.58)	0.544 (0.935)	1.18 (1.78)	0.988 (1.05)	1.01 (1.67)	0.716 (0.996)	
Median [Min, Max]	0.418 [0, 6.67]	0 [0, 3.74]	0.435 [0, 7.66]	0.720 [0, 3.73]	0.435 [0, 7.66]	0.290 [0, 3.74]	
Missing	1 (2.6%)	3 (9.1%)	41 (53.9%)	15 (44.1%)	42 (36.5%)	18 (26.9%)	
IL‐2							0.778
Mean (SD)	1.13 (4.41)	1.52 (4.12)	3.18 (8.47)	1.90 (2.81)	2.11 (6.70)	1.67 (3.64)	
Median [Min, Max]	0 [0, 26.0]	0 [0, 19.5]	0 [0, 35.1]	0.725 [0, 9.05]	0 [0, 35.1]	0 [0, 19.5]	
Missing	1 (2.6%)	3 (9.1%)	41 (53.9%)	15 (44.1%)	42 (36.5%)	18 (26.9%)	
IL‐4							0.829
Mean (SD)	7.61 (9.95)	7.10 (9.53)	13.1 (9.06)	13.5 (10.1)	10.3 (9.87)	9.58 (10.1)	
Median [Min, Max]	4.40 [0, 54.2]	4.24 [0.415, 48.5]	9.64 [0, 41.0]	9.82 [0.365, 34.1]	7.54 [0, 54.2]	6.24 [0.365, 48.5]	
Missing	1 (2.6%)	3 (9.1%)	41 (53.9%)	15 (44.1%)	42 (36.5%)	18 (26.9%)	
IL‐5							0.604
Mean (SD)	0.599 (0.850)	0.962 (1.69)	2.54 (4.85)	1.99 (1.79)	1.53 (3.52)	1.36 (1.78)	
Median [Min, Max]	0.113 [0, 2.81]	0 [0, 6.64]	0.680 [0, 18.1]	1.50 [0, 6.88]	0.460 [0, 18.1]	0.490 [0, 6.88]	
Missing	1 (2.6%)	3 (9.1%)	41 (53.9%)	15 (44.1%)	42 (36.5%)	18 (26.9%)	
IL‐6							0.910
Mean (SD)	9.27 (14.8)	11.9 (19.4)	9.02 (9.85)	72.4 (245)	9.15 (12.6)	35.4 (154)	
Median [Min, Max]	1.75 [0, 59.9]	3.61 [0, 86.2]	5.28 [0, 27.5]	3.32 [0, 1070]	2.88 [0, 59.9]	3.52 [0, 1070]	
Missing	1 (2.6%)	3 (9.1%)	41 (53.9%)	15 (44.1%)	42 (36.5%)	18 (26.9%)	
IL‐8 (CXCL8)							0.512
Mean (SD)	3.16 (6.64)	2.26 (2.24)	1.33 (1.17)	4.08 (10.3)	2.28 (4.92)	2.96 (6.61)	
Median [Min, Max]	0.600 [0, 30.9]	1.47 [0, 9.57]	1.04 [0, 4.23]	0.940 [0, 45.8]	0.930 [0, 30.9]	1.31 [0, 45.8]	
Missing	1 (2.6%)	3 (9.1%)	41 (53.9%)	15 (44.1%)	42 (36.5%)	18 (26.9%)	
IL‐10							0.164
Mean (SD)	0.204 (0.495)	0.985 (4.02)	0.751 (1.32)	0.768 (2.07)	0.466 (1.01)	0.901 (3.37)	
Median [Min, Max]	0 [0, 2.38]	0 [0, 22.0]	0.420 [0, 7.04]	0.0750 [0, 9.13]	0 [0, 7.04]	0 [0, 22.0]	
Missing	1 (2.6%)	3 (9.1%)	41 (53.9%)	15 (44.1%)	42 (36.5%)	18 (26.9%)	
IL‐12p70							0.122
Mean (SD)	1.97 (4.09)	0.952 (1.41)	1.32 (1.98)	1.15 (1.28)	1.66 (3.25)	1.03 (1.35)	
Median [Min, Max]	0.738 [0, 17.5]	0.233 [0, 5.84]	0.485 [0, 8.14]	0.695 [0, 4.69]	0.730 [0, 17.5]	0.540 [0, 5.84]	
Missing	1 (2.6%)	3 (9.1%)	41 (53.9%)	15 (44.1%)	42 (36.5%)	18 (26.9%)	
IL‐13							0.067
Mean (SD)	4.33 (10.5)	1.44 (3.89)	1.45 (3.06)	0.538 (0.951)	2.95 (7.96)	1.09 (3.11)	
Median [Min, Max]	0 [0, 48.9]	0 [0, 19.7]	0 [0, 12.0]	0.150 [0, 3.58]	0 [0, 48.9]	0 [0, 19.7]	
Missing	1 (2.6%)	3 (9.1%)	41 (53.9%)	15 (44.1%)	42 (36.5%)	18 (26.9%)	
IL‐17A (CTLA‐8)							0.546
Mean (SD)	0.913 (2.84)	0.597 (0.916)	1.23 (2.42)	0.871 (1.01)	1.06 (2.63)	0.703 (0.951)	
Median [Min, Max]	0.135 [0, 17.3]	0.190 [0, 3.90]	0.350 [0, 13.2]	0.520 [0, 3.51]	0.345 [0, 17.3]	0.390 [0, 3.90]	
Missing	1 (2.6%)	3 (9.1%)	41 (53.9%)	15 (44.1%)	42 (36.5%)	18 (26.9%)	
IL‐18							0.425
Mean (SD)	103 (118)	83.3 (110)	58.8 (30.8)	83.1 (122)	81.7 (89.9)	83.2 (114)	
Median [Min, Max]	51.4 [11.9, 533]	54.8 [14.1, 619]	55.8 [0, 128]	42.5 [18.8, 551]	54.0 [0, 533]	49.6 [14.1, 619]	
Missing	1 (2.6%)	3 (9.1%)	41 (53.9%)	15 (44.1%)	42 (36.5%)	18 (26.9%)	
IP‐10 (CXCL10)							0.608
Mean (SD)	16.4 (20.6)	18.9 (21.7)	27.9 (16.5)	25.6 (19.6)	21.9 (19.5)	21.5 (21.0)	
Median [Min, Max]	8.80 [1.58, 114]	13.5 [2.80, 93.0]	26.1 [0, 66.9]	19.8 [6.72, 85.6]	15.4 [0, 114]	14.4 [2.80, 93.0]	
Missing	1 (2.6%)	3 (9.1%)	41 (53.9%)	15 (44.1%)	42 (36.5%)	18 (26.9%)	
MCP‐1 (CCL2)							0.418
Mean (SD)	35.6 (30.1)	29.5 (21.8)	36.3 (27.0)	43.8 (46.8)	36.0 (28.5)	35.1 (34.0)	
Median [Min, Max]	25.2 [1.72, 110]	20.2 [5.00, 106]	22.0 [0, 90.8]	33.4 [4.71, 200]	25.1 [0, 110]	22.0 [4.71, 200]	
Missing	1 (2.6%)	3 (9.1%)	41 (53.9%)	15 (44.1%)	42 (36.5%)	18 (26.9%)	
MIP‐1 alpha (CCL3)							0.794
Mean (SD)	0.912 (1.45)	0.792 (1.35)	0.881 (1.79)	1.19 (3.12)	0.897 (1.61)	0.948 (2.19)	
Median [Min, Max]	0.0225 [0, 4.96]	0.205 [0, 5.67]	0.0150 [0, 6.68]	0.0200 [0, 11.2]	0.0150 [0, 6.68]	0.0800 [0, 11.2]	
Missing	1 (2.6%)	3 (9.1%)	41 (53.9%)	15 (44.1%)	42 (36.5%)	18 (26.9%)	
MIP‐1 beta (CCL4)							0.527
Mean (SD)	10.8 (16.4)	8.05 (13.7)	18.3 (21.5)	10.6 (16.8)	14.4 (19.2)	9.05 (14.9)	
Median [Min, Max]	3.83 [0, 70.1]	2.59 [0, 67.1]	6.56 [0, 69.0]	2.51 [0, 51.6]	4.56 [0, 70.1]	2.51 [0, 67.1]	
Missing	1 (2.6%)	3 (9.1%)	41 (53.9%)	15 (44.1%)	42 (36.5%)	18 (26.9%)	
TNF alpha							0.370
Mean (SD)	1.34 (1.51)	1.03 (0.945)	1.40 (1.30)	2.00 (2.04)	1.37 (1.40)	1.40 (1.52)	
Median [Min, Max]	0.865 [0, 8.60]	0.680 [0, 3.61]	0.945 [0, 4.99]	1.18 [0.470, 9.01]	0.890 [0, 8.60]	1.06 [0, 9.01]	
Missing	1 (2.6%)	3 (9.1%)	41 (53.9%)	15 (44.1%)	42 (36.5%)	18 (26.9%)	
TNF beta							0.335
Mean (SD)	0.617 (1.81)	0.242 (0.729)	0.900 (2.16)	0.200 (0.404)	0.753 (1.98)	0.225 (0.618)	
Median [Min, Max]	0 [0, 9.98]	0 [0, 3.34]	0 [0, 10.0]	0 [0, 1.40]	0 [0, 10.0]	0 [0, 3.34]	
Missing	1 (2.6%)	3 (9.1%)	41 (53.9%)	15 (44.1%)	42 (36.5%)	18 (26.9%)	
DPPIV (pg/mL)							0.204
Mean (SD)	1270 (551)	1160 (337)	341 (169)	288 (189)	812 (621)	832 (515)	
Median [Min, Max]	1250 [65.4, 3040]	1180 [567, 1920]	284 [127, 736]	211 [121, 890]	621 [65.4, 3040]	863 [121, 1920]	
Missing	0 (0%)	0 (0%)	38 (50.0%)	14 (41.2%)	38 (33.0%)	14 (20.9%)	
ACE2 (ng/mL)							0.928
Mean (SD)	3.16 (4.67)	3.58 (7.32)	6.61 (11.9)	12.5 (27.6)	5.28 (9.86)	8.16 (20.6)	
Median [Min, Max]	0.918 [0.0160, 17.5]	0.803 [0.0490, 25.5]	1.01 [0.0600, 50.3]	1.18 [0.0210, 104]	0.918 [0.0160, 50.3]	0.984 [0.0210, 104]	
Missing	15 (38.5%)	15 (45.5%)	38 (50.0%)	15 (44.1%)	53 (46.1%)	30 (44.8%)	

### 3.3 DPP‐IV and ACE2 Signature Analysis

Plasma ACE2 and DPP‐IV were measured using ELISA.

DPP‐IV was significantly lower in individuals with COVID‐19 versus those without the infection (322.6 ± 23.1 pg/mL and 1221.2 ± 54.9 pg/mL, respectively; Wilcoxon test *p* < 0.001). In patients with COVID‐19, those with DM had lower DPP‐IV concentrations compared to those without DM, but the difference was not statistically significant (282.3 ± 44.1 pg/mL and 340.8 ± 27.4 pg/mL, respectively; Wilcoxon test *p* value = 0.071).

Plasma ACE2 was not statistically significantly different in individuals with COVID‐19 versus those without (8.6 ± 2.5 pg/mL and 3.3 ± 0.9 pg/mL, respectively; Wilcoxon test *p* = 0.411). In patients with COVID‐19, those with DM had higher plasma ACE2 concentrations versus those without DM, but the difference was not statistically significant (12.5 ± 6.3 pg/mL and 6.6 ± 1.9 pg/mL, respectively; Wilcoxon test *p* value = 0.537).

### 3.4 Clinical Outcomes

Out of 110 patients admitted with COVID‐19, 12 (10.9%) died during hospitalization compared to 3 out of 72 non‐COVID‐19 patients (4.2%). Three (3) out of the 12 COVID‐19 patients who died in the hospital were admitted for shortness of breath (ICD10 R06.02), unspecified fever (ICD‐10 R50.9), or fatigue (ICD‐10 R53.83). The three non‐COVID in‐hospital deaths occurred in patients admitted for pneumonia (ICD‐10 J18.9) or acute respiratory distress (ICD‐10 R06.03).

There was no significant association between DM and COVID‐19 patients' in‐hospital death rate (Supporting Information S5: Table 3), with six DM (17.6%) and six non‐DM (7.9%) COVID‐19 patients dying in hospital (*χ* ^2^ test *p* value = 0.236). Similarly, obesity and BMI were not significant factors associated with in‐hospital death (*p* values of 0.760 and > 0.999, respectively). The odds of in‐hospital death were 21.5 times higher (95% CI = 5.2–88.3, *p* value < 0.001) for the COVID patients admitted to the critical care unit (ICU) compared to those who were not admitted to the ICU. Specifically, seven out of 13 ICU‐admitted patients died in the hospital compared to five deaths occurring in 97 non‐ICU patients.

The odds ratio of in‐hospital death for patients admitted with COVID‐19 versus non‐COVID‐19 patients was not statistically significantly different from 1 (OR = 2.82, 95%CI = 0.86–12.70, *p* value = 0.119). After adjusting for ICU, the association of COVID‐19 diagnoses with death became significant (OR = 6.79, 95%CI = 1.73–36.07, *p* value = 0.012).

COVID‐19 severity was measured on the World Health Organization Original Scale for Clinical Improvement (WHO OSCI) scale (Supporting Information S5: Table 4, Supporting Information S4: Figure 4) [38]. COVID‐19 patients were grouped using the WHO OSCI score into Moderate (score < 5) and severe (>=5 and < 8) disease cohorts. A WHO OSCI score of 8 corresponds to death. At admission, 77 out of 110 COVID patients had a WHO OSCI score of 5 or higher. Notable, five out of the 13 COVID‐19 patients admitted to the ICU had a score of 5 and another eight had a score of 6. At the same time, 64 out of 97 non‐ICU patients (66.0%) scored 5 or above at admission. Additionally, all 12 COVID‐19 patients who died in the hospital had WHO OSCI scores of 5 or 6 at admission, and their scores did not decrease until their death except for a single patient whose score declined from 5 to 4 on Day 3, just before death (Supporting Information S5: Table 3).

On average, COVID‐19 patients were admitted for a slightly shorter duration than non‐COVID patients (mean+/−SEM = 7.3+/−0.9 and 8.8+/−1.1 days, respectively). The patients who died in the hospital were hospitalized for longer times compared to those discharged alive (12.5+/−2.4 vs. 7.5+/−0.7, respectively). The difference between COVID and non‐COVID patients' length of stay was even larger for those who were not discharged on the day of admission (i.e., stayed for more than 1 day), with LOS of 10.1+/−1.2 days for non‐COVID patients discharged alive versus 7.4+/−0.9 days for the COVID patients discharged alive. For the patients who died in the hospital, the LOS were 11.0+/−3.8 and 12.8+/−2.9 for non‐COVID versus COVID patients, respectively.

## 4 Discussion

Our study compares gene expression, protein expression, and clinical outcomes in patients hospitalized with COVID‐19 to non‐COVID‐19 stratified by diabetes. First, we evaluated the differential expression of several genes between the COVID and non‐COVID groups. The relevance of these gene pathways in the pathophysiology of COVID‐19 has been documented in the literature or is biologically plausible. Several gene pathways we identified as differentially expressed in this study contribute to disease progression, such as AXL, BAMBI, CLEC6A, IFI27, Krt8, Nectin‐2, PRKG1, and PDE2A, as previously reported [39, 40, 41, 42]. Below, we summarize the potential role of these genes in COVID‐19. The altered regulation of several other genes was also identified, and their role in COVID‐19 progression remains poorly elucidated. Further studies are required to understand their role in the pathogenesis of the disease.

AXL functions as a tyrosine receptor kinase within the TAM subfamily of receptor tyrosine kinases and functions to control mechanisms of inflammation and coagulation. Like other TAM receptors (Tyro3 and Mer), AXL has important effects on hemostasis and inflammation [43]. TAM subfamily of receptor tyrosine kinases, when activated, have also been demonstrated to reduce the production of cytokines, including type I IFNs, IL‐6, and TNF, following activation of various TLRs, including TLR‐3, 4, and 9 [44]. TLR‐9 is associated with cellular defense against viral infections and is hypothesized to function similarly against COVID‐19; thus, TAM activation may downregulate important cytokine functions in the immune and inflammasome response [45]. Our study demonstrated a differential increase in AXL expression, which may stunt immune responses to COVID‐19 and increase the risk of disease complications. In addition to AXLs role in cytokine production, previous research suggests that AXL may play a role in the entry of SARS‐CoV‐2 virus into human cells along with ACE2, especially given the elevated expression of AXL in comparison to ACE2 in human pulmonary and bronchial tissue [46]. Increased expression of AXL, thus, may predispose specific patients to be more susceptible to COVID‐19 infection.

Another potential mechanism of AXL in the pathogenesis of SARS‐CoV‐2 is described by its role in platelet activation. Mouse models have demonstrated that the binding of growth arrest‐specific gene 6 (Gas6) to AXL receptors contributes to platelet thrombus formation. Similarly, the inhibition of such an interaction inhibits platelet aggregation and degranulation [47]. Thrombosis is commonly seen in SARS‐CoV‐2, and several mechanisms, including inflammation and spike protein‐ACE2 binding, have been cited as platelet activation pathways [48]. Although thrombosis outcomes were not assessed in the patients of this study, the increased AXL expression and activation may similarly contribute to the formation of platelet‐derived thrombosis. Understanding the role of AXL may improve the selection of anticoagulant strategies in this patient population. For example, warfarin blocks Gas6‐mediated AXL activation, while direct oral antithrombotics and heparins do not [49, 50]. Nonetheless, full‐dose anticoagulation in hospitalized patients with COVID‐19 who are not critically ill is recommended by several treatment guidelines without specific recommendations for a particular agent [51]. In critically ill patients, standard prophylaxis is recommended unless the patient's presentation is consistent with thrombosis.

BAMBI, also known as BMP and activin membrane‐bound inhibitor, has been demonstrated to modulate the expression of ACE2 at the mRNA level [42]. When upregulated in cells, BAMBI increases the proportion of COVID‐19‐infected cells [42]. SARS‐CoV‐2 viral entry into human cells has been observed by binding the spike protein to the ACE2, promoting attachment and fusion. SARS‐CoV‐2 has a significantly higher affinity for ACE2 than SARS‐CoV, contributing towards the greater degree of pathogenicity of the newer disease [52]. Increased expression of BAMBI in COVID‐19 patients may indicate underlying susceptibility to viral invasion and infection. BAMBI is also highly expressed in platelets and endothelial cells and has a role in thrombus formation [53].

CLEC6A, also known as dectin‐2, is a member of the C‐type lectins typically expressed on macrophages and dendritic cells as part of C‐type lectin receptors (CLRs). The activation of these receptors is responsible for a myriad of cellular functions, such as cell adhesion, stimulation of endocytosis, tissue repair, and the activation of platelets in the natural immune system [54]. Activating CLRs via CLEC6A (and other pathways) stimulated the recruitment of tyrosine kinases and beta cell lymphoid tissue 10 to form complexes, triggering the NF‐kB and MAPKs pathways [55]. Although the role of CLEC6A is not well elucidated in COVID‐19, some data suggest its relevance in the pathogenesis of MERS‐CoV, a very closely related virus. In MERS‐CoV, the increased activation of CLRs has been shown to contribute towards a more robust immune response and promote viral recognition, triggering a pro‐inflammatory response [56]. It is possible that CLEC6A plays a similar role in COVID‐19 and may contribute to the “cytokine storm” that is often cited as the catalyst for COVID‐19 mortality.

The protein coded by IFI27 was previously shown to be associated with other viral infections, including Hepatitis C, respiratory syncytial virus (RSV) infection, and Enterovirus 71 (EV71) hand, foot, and mouth disease. IFI27 has also been proposed as a biomarker to differentiate between influenza and bacterial respiratory infection, although its ability to distinguish between different viruses is limited [57]. More recently, IFI27 has been proposed as a biomarker for an early prediction of COVID‐19 outcomes [41]. IFI27 counteracts innate immune responses and has a positive effect on SARS‐CoV‐2 replication [58]. Therefore, mechanistic evidence suggests that elevated IFI27 leads to elevated SARS‐CoV‐2 viral load. While there is conflicting evidence on viral load and COVID‐19 outcomes, older individuals with higher viral loads had worse outcomes, and overall transmissibility increases as viral load increases [59].

KRT8 is a gene expressed by transitional alveolar epithelial cells during lung injury. Type 1 and type 2 alveolar epithelial cells constitute the functioning lung parenchyma. Following an injury event, the lung will undergo a recovery process that involves the proliferation of type 2 alveolar epithelial cells, which will then undergo a transitional state and fully differentiate into type 1 alveolar epithelial cells. This transitional state is characterized by the expression of specific gene signatures, including Krt8 [60]. Several studies have shown that Krt8+ transitional cells are abundant in patients with COVID‐19. In lethal cases, however, it has been shown that the increase in Krt8+ is not matched by an increase in type 1 alveolar epithelial cells, indicating a disrupting differentiation process and suggesting that regenerative processes are impaired in the COVID‐19 disease state [60, 61]. Chronically, Krt8 may also be implicated in the development of fibrotic patterns following COVID‐19 infection. In bleomycin‐induced models of pulmonary fibrosis, Krt8+ cells showed increased expression of pro‐fibrotic proteins such as Areg and Hbegf and the presence of myofibroblasts [62]. Examining gene profiles in non‐resolvable COVID‐19 revealed increases in fibrotic gene and Krt8 expression similar to those in idiopathic pulmonary fibrosis [63]. Severe COVID‐19 has been characterized by the development of fibrosis with increased collagen deposition, supporting the proposed fibrotic pathological process [64, 65].

NECTIN‐2 is a member of the nectin family involved in cellular adhesion molecules (CAMs), which regulate critical cell‐cell interactions. One of these interactions is with the DNAX accessory molecule 1 (DNAM‐1), which mediates the activation of natural killer cells and cytotoxic T cells [66]. A study investigating the expression of natural killer cell ligands and receptors in COVID‐19 found an increased proportion of activated natural killer cells in moderate to severe COVID‐19. Paradoxically, however, there was a decrease in the amount of the activating DNAM‐1 receptor despite an increase in nectin‐2 expression [67]. This response is hypothesized to be a stress‐induced downregulation in which high‐stress environments promote receptor endocytosis and subsequent lysosome degradation. The loss of DNAM‐1 receptors eventually impairs the function of natural killer cells [68]. Thus, the high activity of nectin‐2 in patients with COVID‐19 is primarily a response to the initial infection, but overactivity may ultimately stunt long‐term natural killer cell effectiveness.

PRKG1, also known as cGMP‐dependent protein kinase 1, phorphoryles many targets, regulating functions such as platelet activation and adhesion and cardiomyocyte cGMP [69, 70]. Within PRKG1, however, is an ELDKY gene motif that demonstrates strong molecular mimicry to the COVID‐19 spike protein [71]. ELDKY has been seen to elicit antibody responses following COVID‐19 immunization with the spike protein mRNA vaccine, and increased antibody response to ELDKY has been observed in patients with severe COVID‐19 [72, 73]. It is hypothesized that the cross‐reactivity of the ELDKY motif and the spike protein may be responsible for antibody‐mediated effects on both platelet and cardiac function seen in COVID‐19 [48, 71]. Another study examining genomic data of COVID‐19 patients found that PRKG1 alleles are also linked with increased mortality in the middle‐aged European‐American population (ages 45–54). However, the definitive mechanism of this risk factor remains unknown [74].

Phosphodiesterases (PDEs) play important roles in hydrolyzing and inactivating cAMP and cGMP in cellular processes. PDE2A, however, is stimulated explicitly by cGMP to hydrolyze cAMP preferentially [75]. These intracellular cyclic nucleotides have been known to play a role in maintaining the endothelial cell barrier [76]. Although little data specifically connecting PDE2A to COVID‐19 exists, this gene has been previously implicated in the development of lung injury. In mouse studies, PDE2A has downregulated lung nitric oxide synthase (NOS) in early‐stage injury, thus promoting alveolar inflammation and lung injury [77]. In late‐stage injury, however, PDE2A inhibits macrophage NOS expression, which has been suggested to promote lung injury resolution following initial insult [75]. The mechanism of this pathological process is hypothesized to involve pulmonary endothelial barrier dysfunction caused by decreases in cAMP catalyzed by increased PDE2A expression [78]. A separate study evaluating the effects of tumor necrosis factor‐α (TNF‐α) found an upregulation in PDE2A downstream and increased membrane permeability. PDE2 inhibition in mice lungs reduced the wet‐to‐dry ratio and albumin movement, demonstrating minimized fluid translocation [79]. PDE2A's role in developing lung injury through disrupted endothelial membrane barriers likely reflects the upregulation of this gene in COVID‐19 patients and the increased risk for pulmonary complications such as acute respiratory distress syndrome (ARDS).

In addition to gene expression, we measured a panel of cytokines, chemokines, circulating ACE2, and circulating DPP‐IV. While we did not find significant differences in TNF and IL‐6 between groups, individuals with COVID‐19 and DM did have numerically higher values than those without either disease. Previous literature has highlighted elevated cytokines and chemokines leading to a “cytokine storm” in individuals with severe COVID‐19 [80]. Notably, nearly 80% of patients in the current study received dexamethasone, while others received alternative corticosteroids. These drugs are frequently administered at the earliest sign of severe illness to prevent cytokine storm [81]. As such, drug therapy may have influenced the relatively small changes in cytokines and chemokines. In addition, individuals with DM were more likely to receive tocilizumab, an IL‐6‐directed monoclonal antibody.

Several genes, including GRASP, KRT8, MYZAP, PRKG1, and SMIM24 were differentially expressed in the COVID versus no COVID and diabetes versus no diabetes subgroup analyses. Although poorly understood, the gene expression pattern potentially gives insight into the inflammatory changes observed in the COVID‐19 positive groups. PRKG1, as noted above, functions cGMP protein kinase in multiple cell types. An in vitro study in T lymphocytes demonstrated that PRKG1 inhibits cellular proliferation and production of IL‐2 [82]. GRASP, short for Grp‐1 associated scaffold protein, also known as Tamalin, shares significant homology with Cybr, another scaffold protein strongly expressed by the immune system, connecting to T lymphocyte cellular adhesion and function [83]. Previous in vitro studies have shown that Cybr is upregulated by increased levels of IL‐2 [84]. Thus, the decreased IL‐2 levels, potentially driven by changes in PRKG1, seen in individuals with diabetes and COVID‐19 may impair this immune mechanism, predisposing individuals with diabetes to worsened immune responses. KRT8, in addition to its fibrotic properties, has been documented to cause a blunted response of the tumor necrosis factor (TNF)‐associated factor 6 (TRAF6), responsible for the production of pro‐inflammatory cytokines, such as IL‐6 [85]. Paradoxically, however, our study results indicated that the increased levels of KRT8 in individuals with diabetes and COVID‐19 correlated with higher levels of IL‐6. More research on this interaction is needed to fully understand the nature of this pathway in vivo. As far as we are aware, this study is the first to identify MYZAP and SMIM24 as potential gene regulators in an infectious‐immune response. Future studies may elucidate the role of these genes in the context of inflammatory changes.

The function of these five genes in the development of diabetes may explain the connection to immune changes. Krt8 has been documented in β‐cell dysfunction. β‐cell Krt8 knockout mice demonstrated increased cellular fragility and smaller mitochondria. These mice also showed decreased insulin production in response to glucose stimulation, ultimately suggesting Krt8's role in insulin deficiency [86]. Presently, however, no other literature exists regarding the remaining four critically differentially expressed genes, GRASP, MYZAP, PRKG1, and SMIM24, in diabetes patients.

Current literature on gene expression in diabetes highlights alternative gene patterns. A study analyzing gene expression between individuals with type II diabetes and controls found differential expression of the RIG‐I‐like receptor signaling pathway, highlighting the genes TBK1, ATG5, TANK, IL8, and MAVS [87]. This pathway has also been previously implicated in COVID‐19 and the ability of SARS‐CoV‐2 to avoid the innate immune response [88]. Another study investigating differential monocyte gene expression in individuals with diabetes identified IRF1, GATA6, SP11, EPAS1, NFKB2, and STATB3 as key affected transcription factors. These genes function in both metabolic pathway control and immunity, suggesting a connection to diabetes pathogenesis [89].

Serum ACE2 is associated with more severe COVID‐19 infection [90, 91]. Similarly, circulating DPP‐IV has been reported to be lower in individuals with severe COVID‐19 [92, 93]. Our findings affirm these previous observations.

Finally, clinical outcomes were assessed, and we found an increased mortality in patients with COVID‐19 and concomitant diabetes compared to patients with COVID‐19 alone (17.6% vs. 7.9%); however, the difference did not reach statistical significance. The study was not designed to evaluate clinical outcomes and likely had insufficient power to detect significant differences.

The strengths of this study include the sample size and the quality of sample collection. Furthermore, the study utilized whole blood, which likely reflects a more accurate representation of gene expression relative to samples from other sources, such as nasal swabs. In addition, stratified analysis by individuals with and without diabetes allowed focus on a high‐risk population. Limitations include using leftover clinical specimens, which may have been available for only some subjects in the study. Nonetheless, this strategy allowed for the performance of the study without placing unnecessary burdens on patients for additional blood collection. The retrospective nature of data collection limited the clinical laboratory values available for each subject. Regardless of these limitations, the study provides critical insight into the altered gene expression patterns in individuals with COVID‐19 with and without diabetes mellitus.

## 5 Conclusions

Gene expression is altered in individuals with and without DM who have COVID‐19. Many differentially expressed genes are involved in the disease process and represent potential drug targets.

## Author Contributions

Luigi Brunetti, Ah‐Ng Kong, and Ronald G. Nahass designed the study. Luigi Brunetti, Wael Hassan, Andrew Wassef, and Marshall Yuan performed data acquisition and editing. Davit Sargsyan, Vahe Nersisyan, and Javier Cabrera performed statistical analysis and data visualization. All authors were involved in drafting the original manuscript. All authors satisfy the criteria for authorship. All authors have read and agreed to the published version of the manuscript.

## Ethics Statement

The Institutional Review Board of Robert Wood Johnson University Hospital Somerset reviewed and approved the study (IRB20‐16).

## Conflicts of Interest

Davit Sargsyan is an employee of Johnson and Johnson. The opinions expressed in this paper are those of the authors and do not necessarily represent those of Johnson and Johnson.

## Supporting information


**Supplemental Figure 1.** Graphical representation of patient baseline demographic clinical characteristics.

**Supplemental Figure 2**. Number of genes evaluated versus number of hits per gene in the total sample.

**Supplemental Figure 3.** Graphical representation of cytokines and chemokines.

**Supplemental Figure 4.** Comparison of frequency of WHO OSCI over time between individuals with and without diabetes mellitus (DM).

Supporting Table 1. Available patient clinical laboratory values upon hospital presentation. Supporting Table 2. Comparison of medication usage between patient with and without COVID‐19 and diabetes. Supporting Table 3. Patient hospital disposition after admission. Supporting Material 4. Comparison of disease progression with the WHO Ordinal Scale for Clinical Severity.

## Data Availability

The data that support the findings of this study are available from the corresponding author upon reasonable request.
