# Supplementary figures and images for: Genetic and Inflammatory Signatures Associated With Worse Prognosis in Hospitalized Patients With Severe SARS‐CoV‐2 Infection With and Without Diabetes
Source: J Med Virol. 2025 Jun 6;97(6):e70425. doi: 10.1002/jmv.70425 (PMC12143195; doi:10.1002/jmv.70425)

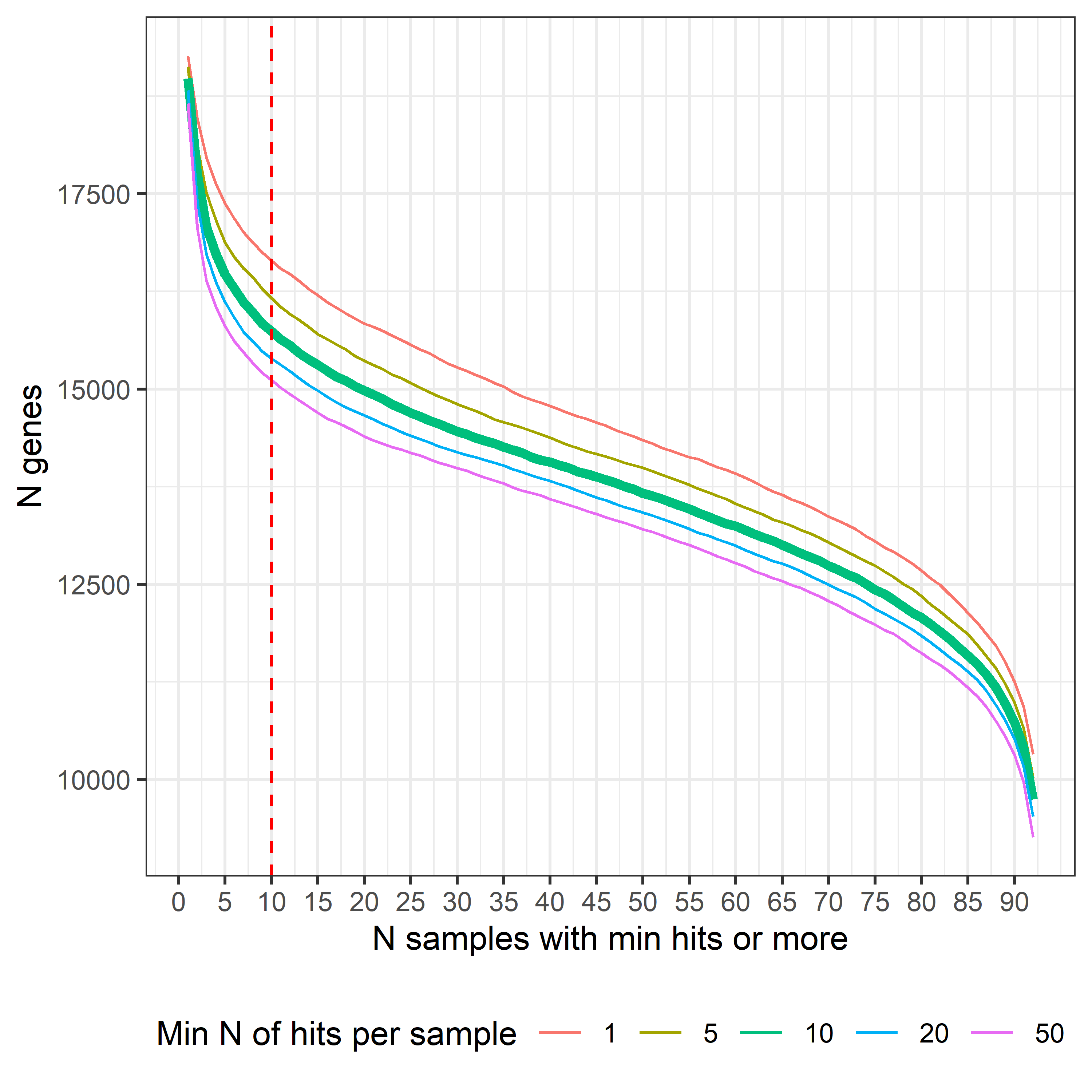

Supplement: Supplementary file 2 — Supplemental Figure 2. Number of genes evaluated versus number of hits per gene in the total sample. [file JMV-97-e70425-s004.tiff]

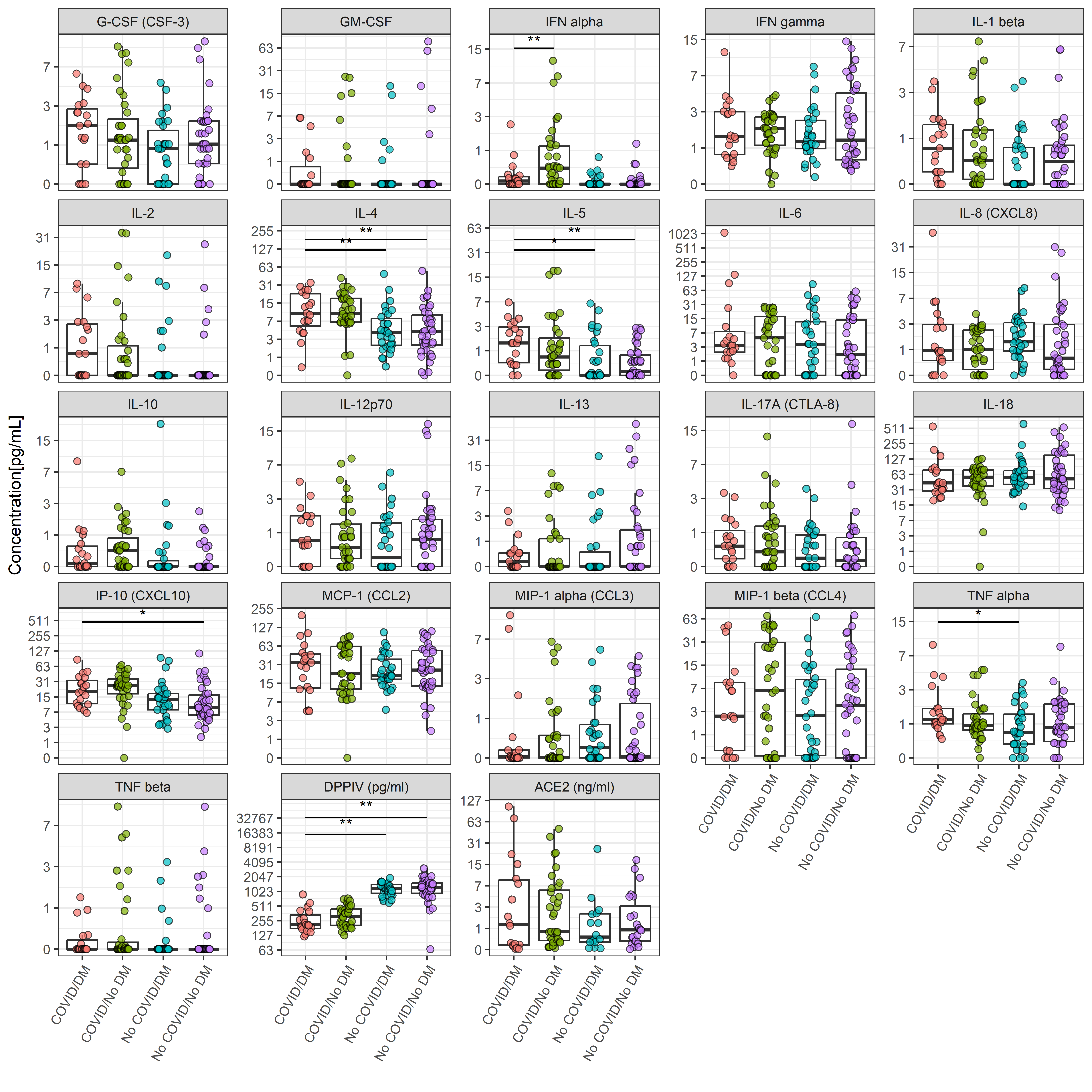

Supplement: Supplementary file 3 — Supplemental Figure 3. Graphical representation of cytokines and chemokines. [file JMV-97-e70425-s001.tiff]

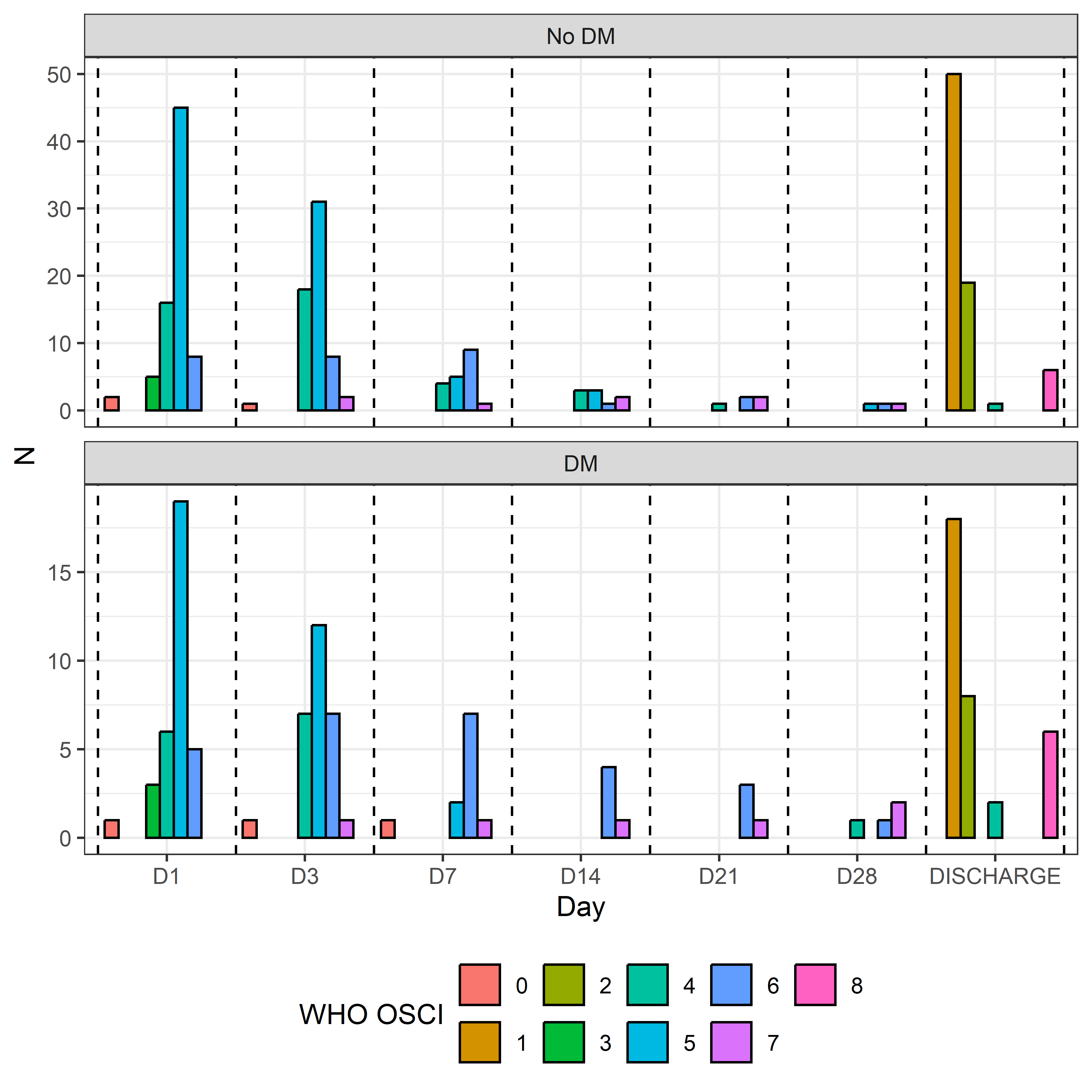

Supplement: Supplementary file 4 — Supplemental Figure 4. Comparison of frequency of WHO OSCI over time between individuals with and without diabetes mellitus (DM). [file JMV-97-e70425-s003.tiff]
