# Supplementary material for: Genetic and Inflammatory Signatures Associated With Worse Prognosis in Hospitalized Patients With Severe SARS‐CoV‐2 Infection With and Without Diabetes
Source: J Med Virol. 2025 Jun 6;97(6):e70425. doi: 10.1002/jmv.70425 (PMC12143195; doi:10.1002/jmv.70425)
Supplement: Supplementary file 5 — Supporting Table 1. Available patient clinical laboratory values upon hospital presentation. Supporting Table 2. Comparison of medication usage between patient with and without COVID‐19 and diabetes. Supporting Table 3. Patient hospital disposition after admission. Supporting Material 4. Comparison of disease progression with the WHO Ordinal Scale for Clinical Severity. [file JMV-97-e70425-s005.docx]

Supplementary Tables

| Supplementary Table 1. Available patient clinical laboratory values upon hospital presentation | | | | | | | p-Value |
| --- | --- | --- | --- | --- | --- | --- | --- |
|  | **No Covid** | | **COVID** | | **Overall** | |  |
|  | No DM  **(N=39)** | Any DM  **(N=33)** | No DM  **(N=76)** | Any DM  **(N=34)** | No DM  **(N=115)** | Any DM  **(N=67)** |  |
| HbA1c |  |  |  |  |  |  | <0.001 |
| Mean (SD) | 5.98 (0.911) | 8.85 (2.57) | 6.21 (0.586) | 8.48 (2.48) | 6.08 (0.779) | 8.66 (2.50) |  |
| Median [Min, Max] | 5.60 [4.80, 8.80] | 8.50 [5.40, 13.1] | 6.30 [5.50, 7.50] | 7.80 [6.00, 14.3] | 5.90 [4.80, 8.80] | 7.95 [5.40, 14.3] |  |
| Missing | 22 (56.4%) | 14 (42.4%) | 62 (81.6%) | 15 (44.1%) | 84 (73.0%) | 29 (43.3%) |  |
| Serum vitamin D |  |  |  |  |  |  | 0.154 |
| Mean (SD) | NA (NA) | 21.6 (6.94) | 83.1 (13.5) | 66.2 (73.5) | 83.1 (13.5) | 48.3 (57.5) |  |
| Median [Min, Max] | NA [NA, NA] | 21.6 [16.7, 26.5] | 88.0 [63.5, 93.0] | 36.0 [12.6, 150] | 88.0 [63.5, 93.0] | 26.5 [12.6, 150] |  |
| Missing | 39 (100%) | 31 (93.9%) | 72 (94.7%) | 31 (91.2%) | 111 (96.5%) | 62 (92.5%) |  |
| ALT |  |  |  |  |  |  | 0.969 |
| Mean (SD) | 22.6 (10.6) | 23.3 (31.7) | 46.7 (49.6) | 73.7 (165) | 38.8 (42.5) | 48.9 (121) |  |
| Median [Min, Max] | 20.0 [8.00, 49.0] | 15.0 [7.00, 186] | 31.0 [8.00, 286] | 29.0 [8.00, 915] | 27.0 [8.00, 286] | 22.0 [7.00, 915] |  |
| Missing | 3 (7.7%) | 1 (3.0%) | 3 (3.9%) | 1 (2.9%) | 6 (5.2%) | 2 (3.0%) |  |
| AST |  |  |  |  |  |  | 0.888 |
| Mean (SD) | 26.3 (20.8) | 22.5 (22.0) | 55.0 (53.7) | 96.6 (241) | 45.3 (47.2) | 60.1 (175) |  |
| Median [Min, Max] | 19.5 [11.0, 118] | 16.5 [9.00, 134] | 39.0 [11.0, 325] | 36.0 [14.0, 1350] | 32.5 [11.0, 325] | 26.0 [9.00, 1350] |  |
| Missing | 1 (2.6%) | 1 (3.0%) | 2 (2.6%) | 1 (2.9%) | 3 (2.6%) | 2 (3.0%) |  |
| hs-CRP |  |  |  |  |  |  | 0.257 |
| Mean (SD) | 0.0400 (NA) | NA (NA) | 9.00 (7.31) | 9.14 (7.68) | 8.44 (7.41) | 9.14 (7.68) |  |
| Median [Min, Max] | 0.0400 [0.0400, 0.0400] | NA [NA, NA] | 10.1 [0.118, 23.3] | 7.75 [1.55, 25.0] | 8.23 [0.0400, 23.3] | 7.75 [1.55, 25.0] |  |
| Missing | 38 (97.4%) | 33 (100%) | 61 (80.3%) | 26 (76.5%) | 99 (86.1%) | 59 (88.1%) |  |
| CRP |  |  |  |  |  |  | 0.054 |
| Mean (SD) | 2.45 (2.82) | 14.5 (9.31) | 9.49 (10.2) | 5.15 (3.27) | 7.73 (9.35) | 9.30 (7.88) |  |
| Median [Min, Max] | 1.63 [0.0300, 6.52] | 14.8 [2.79, 25.5] | 5.73 [0.300, 33.1] | 5.53 [0.590, 8.74] | 3.87 [0.0300, 33.1] | 7.54 [0.590, 25.5] |  |
| Missing | 35 (89.7%) | 29 (87.9%) | 64 (84.2%) | 29 (85.3%) | 99 (86.1%) | 58 (86.6%) |  |
| Fibrinogen |  |  |  |  |  |  | 0.001 |
| Mean (SD) | 357 (267) | 986 (NA) | 539 (156) | 539 (113) | 532 (161) | 561 (147) |  |
| Median [Min, Max] | 357 [168, 546] | 986 [986, 986] | 509 [269, 887] | 567 [218, 697] | 509 [168, 887] | 574 [218, 986] |  |
| Missing | 37 (94.9%) | 32 (97.0%) | 29 (38.2%) | 14 (41.2%) | 66 (57.4%) | 46 (68.7%) |  |
| D-Dimer |  |  |  |  |  |  | 0.981 |
| Mean (SD) | 1260 (1380) | 1270 (1160) | 436 (962) | 722 (959) | 529 (1040) | 777 (973) |  |
| Median [Min, Max] | 662 [322, 4350] | 1010 [267, 2540] | 17.6 [0.320, 7290] | 559 [0.210, 4330] | 322 [0.320, 7290] | 577 [0.210, 4330] |  |
| Missing | 31 (79.5%) | 30 (90.9%) | 13 (17.1%) | 7 (20.6%) | 44 (38.3%) | 37 (55.2%) |  |
| ESR |  |  |  |  |  |  | 0.171 |
| Mean (SD) | 25.5 (27.4) | 50.3 (29.4) | 27.7 (21.3) | 43.6 (34.8) | 27.3 (21.8) | 45.8 (32.4) |  |
| Median [Min, Max] | 19.5 [3.00, 60.0] | 44.5 [22.0, 101] | 24.0 [1.00, 80.0] | 31.5 [1.00, 115] | 24.0 [1.00, 80.0] | 37.0 [1.00, 115] |  |
| Missing | 35 (89.7%) | 27 (81.8%) | 57 (75.0%) | 22 (64.7%) | 92 (80.0%) | 49 (73.1%) |  |
| Ferritin |  |  |  |  |  |  | 0.636 |
| Mean (SD) | 214 (234) | 691 (928) | 1020 (1190) | 1490 (2890) | 967 (1170) | 1310 (2600) |  |
| Median [Min, Max] | 169 [27.0, 607] | 283 [16.1, 2880] | 541 [18.7, 5750] | 305 [116, 12700] | 516 [18.7, 5750] | 297 [16.1, 12700] |  |
| Missing | 34 (87.2%) | 24 (72.7%) | 6 (7.9%) | 2 (5.9%) | 40 (34.8%) | 26 (38.8%) |  |
| LDH |  |  |  |  |  |  | 0.139 |
| Mean (SD) | 485 (404) | 298 (100) | 340 (170) | 326 (160) | 348 (187) | 323 (153) |  |
| Median [Min, Max] | 325 [209, 1080] | 268 [219, 438] | 293 [148, 890] | 286 [142, 824] | 293 [148, 1080] | 286 [142, 824] |  |
| Missing | 35 (89.7%) | 29 (87.9%) | 6 (7.9%) | 3 (8.8%) | 41 (35.7%) | 32 (47.8%) |  |
| Lactic Acid |  |  |  |  |  |  | 0.969 |
| Mean (SD) | 1.99 (1.76) | 2.26 (1.77) | 5.86 (21.4) | 9.33 (30.8) | 4.79 (18.2) | 6.30 (23.4) |  |
| Median [Min, Max] | 1.70 [0.700, 8.50] | 1.65 [1.00, 7.50] | 1.30 [0.700, 118] | 1.60 [0.800, 150] | 1.40 [0.700, 118] | 1.60 [0.800, 150] |  |
| Missing | 21 (53.8%) | 15 (45.5%) | 29 (38.2%) | 10 (29.4%) | 50 (43.5%) | 25 (37.3%) |  |
| Procalcitonin |  |  |  |  |  |  | 0.254 |
| Mean (SD) | 0.944 (2.00) | 3.04 (5.76) | 0.932 (4.02) | 0.430 (0.953) | 0.933 (3.84) | 1.25 (3.46) |  |
| Median [Min, Max] | 0.157 [0.0340, 5.02] | 0.474 [0.0510, 18.9] | 0.130 [0.0400, 27.7] | 0.160 [0.0300, 4.65] | 0.130 [0.0340, 27.7] | 0.190 [0.0300, 18.9] |  |
| Missing | 33 (84.6%) | 21 (63.6%) | 27 (35.5%) | 8 (23.5%) | 60 (52.2%) | 29 (43.3%) |  |
| Plasma glucose |  |  |  |  |  |  | <0.001 |
| Mean (SD) | 122 (34.9) | 226 (107) | 118 (26.1) | 204 (96.2) | 120 (29.3) | 215 (101) |  |
| Median [Min, Max] | 110 [82.0, 215] | 198 [97.0, 515] | 112 [84.0, 257] | 173 [111, 498] | 112 [82.0, 257] | 189 [97.0, 515] |  |
| Missing | 0 (0%) | 1 (3.0%) | 1 (1.3%) | 1 (2.9%) | 1 (0.9%) | 2 (3.0%) |  |
| Serum creatinine |  |  |  |  |  |  | 0.866 |
| Mean (SD) | 1.15 (0.892) | 1.41 (0.945) | 2.39 (9.78) | 1.96 (3.22) | 1.96 (7.95) | 1.69 (2.39) |  |
| Median [Min, Max] | 0.843 [0.429, 5.06] | 1.07 [0.484, 3.84] | 0.850 [0.290, 82.0] | 0.860 [0.500, 18.0] | 0.850 [0.290, 82.0] | 0.960 [0.484, 18.0] |  |
| Missing | 0 (0%) | 1 (3.0%) | 1 (1.3%) | 1 (2.9%) | 1 (0.9%) | 2 (3.0%) |  |

| Supplementary Table 2. Comparison of medication usage between patient with and without COVID-19 and diabetes | | | | | | | |
| --- | --- | --- | --- | --- | --- | --- | --- |
|  | **No Covid** | | **COVID** | | **Overall** | | p-Value |
|  | No DM  **(N=39)** | Any DM  **(N=33)** | No DM  **(N=76)** | Any DM  **(N=34)** | No DM  **(N=115)** | Any DM  **(N=67)** |  |
| Tocilizumab | 0 (0%) | 0 (0%) | 6 (7.9%) | 6 (17.6%) | 6 (5.2%) | 6 (9.0%) | 0.007 |
| Remdesivir | 0 (0%) | 0 (0%) | 55 (72.4%) | 26 (76.5%) | 55 (47.8%) | 26 (38.8%) | <0.001 |
| Dexamethasone | 6 (15.4%) | 4 (12.1%) | 58 (76.3%) | 27 (79.4%) | 64 (55.7%) | 31 (46.3%) | <0.001 |
| Methylprednisolone | 5 (12.8%) | 3 (9.1%) | 3 (3.9%) | 2 (5.9%) | 8 (7.0%) | 5 (7.5%) | 0.343 |
| Prednisone | 8 (20.5%) | 1 (3.0%) | 1 (1.3%) | 0 (0%) | 9 (7.8%) | 1 (1.5%) | <0.001 |
| Hydrocortisone | 0 (0%) | 0 (0%) | 1 (1.3%) | 3 (8.8%) | 1 (0.9%) | 3 (4.5%) | 0.032 |
| Azithromycin | 3 (7.7%) | 1 (3.0%) | 5 (6.6%) | 0 (0%) | 8 (7.0%) | 1 (1.5%) | 0.379 |
| Hydroxychloroquine | 1 (2.6%) | 1 (3.0%) | 7 (9.2%) | 1 (2.9%) | 8 (7.0%) | 2 (3.0%) | 0.324 |
| ACEi | 2 (5.1%) | 8 (24.2%) | 6 (7.9%) | 7 (20.6%) | 8 (7.0%) | 15 (22.4%) | 0.023 |
| ARBs | 7 (17.9%) | 6 (18.2%) | 9 (11.8%) | 5 (14.7%) | 16 (13.9%) | 11 (16.4%) | 0.77 |
| ARNI | 2 (5.1%) | 0 (0%) | 0 (0%) | 2 (5.9%) | 2 (1.7%) | 2 (3.0%) | 0.104 |
| Insulin | 11 (28.2%) | 30 (90.9%) | 12 (15.8%) | 28 (82.4%) | 23 (20.0%) | 58 (86.6%) | <0.001 |
| Metformin | 0 (0%) | 5 (15.2%) | 1 (1.3%) | 6 (17.6%) | 1 (0.9%) | 11 (16.4%) | 0.001 |
| Glimepiride | 1 (2.6%) | 3 (9.1%) | 0 (0%) | 3 (8.8%) | 1 (0.9%) | 6 (9.0%) | 0.047 |
| Glipizide | 0 (0%) | 2 (6.1%) | 0 (0%) | 3 (8.8%) | 0 (0%) | 5 (7.5%) | 0.026 |
| Sitagliptin | 0 (0%) | 3 (9.1%) | 0 (0%) | 4 (11.8%) | 0 (0%) | 7 (10.4%) | 0.005 |
| Full Dose Anticoagulation | 25 (64.1%) | 27 (81.8%) | 61 (80.3%) | 29 (85.3%) | 86 (74.8%) | 56 (83.6%) | 0.114 |
| Prophylactic Anticoagulation | 21 (53.8%) | 16 (48.5%) | 66 (86.8%) | 28 (82.4%) | 87 (75.7%) | 44 (65.7%) | <0.001 |

| Supplementary Table 3. Patient hospital disposition after admission | | | | | | | |
| --- | --- | --- | --- | --- | --- | --- | --- |
|  | **No Covid** | | **COVID** | | **Overall** | | p-Value |
|  | No DM  **(N=39)** | Any DM  **(N=33)** | No DM  **(N=76)** | Any DM  **(N=34)** | No DM  **(N=115)** | Any DM  **(N=67)** |  |
| Critical Care | 14 (35.9%) | 6 (18.2%) | 6 (7.9%) | 7 (20.6%) | 20 (17.4%) | 13 (19.4%) | 0.003 |
| ICU LOS |  |  |  |  |  |  | 0.309 |
| Mean (SD) | 2.90 (7.36) | 1.55 (4.40) | 0.553 (2.50) | 2.91 (8.60) | 1.35 (4.84) | 2.24 (6.84) |  |
| Median [Min, Max] | 0 [0, 43.0] | 0 [0, 20.0] | 0 [0, 19.0] | 0 [0, 47.0] | 0 [0, 43.0] | 0 [0, 47.0] |  |
| LOS |  |  |  |  |  |  | 0.553 |
| Mean (SD) | 8.21 (9.80) | 9.48 (8.61) | 6.70 (7.91) | 8.71 (11.1) | 7.21 (8.58) | 9.09 (9.89) |  |
| Median [Min, Max] | 4.00 [0, 46.0] | 6.00 [0, 34.0] | 4.00 [0, 41.0] | 4.00 [0, 53.0] | 4.00 [0, 46.0] | 6.00 [0, 53.0] |  |
| Expired | 2 (5.1%) | 1 (3.0%) | 6 (7.9%) | 6 (17.6%) | 8 (7.0%) | 7 (10.4%) | 0.129 |
|  | | | | | | |  |

| Supplementary Material 4. Comparison of disease progression with the WHO Ordinal Scale for Clinical Severity | | | |
| --- | --- | --- | --- |
|  | COVID/No DM  (N=76) | COVID/Any DM  (N=34) | p-Value |
| WHO-OSCI D1 |  |  | 0.951 |
| 0 | 2 (2.6%) | 1 (2.9%) |  |
| 3 | 5 (6.6%) | 3 (8.8%) |  |
| 4 | 16 (21.1%) | 6 (17.6%) |  |
| 5 | 45 (59.2%) | 19 (55.9%) |  |
| 6 | 8 (10.5%) | 5 (14.7%) |  |
| 7 | 0 (0%) | 0 (0%) |  |
| WHO-OSCI D3 |  |  | 0.680 |
| 0 | 1 (1.3%) | 1 (2.9%) |  |
| 4 | 18 (23.7%) | 7 (20.6%) |  |
| 5 | 31 (40.8%) | 12 (35.3%) |  |
| 6 | 8 (10.5%) | 7 (20.6%) |  |
| 7 | 2 (2.6%) | 1 (2.9%) |  |
| Missing | 16 (21.1%) | 6 (17.6%) |  |
| WHO-OSCI D7 |  |  | 0.315 |
| 0 | 0 (0%) | 1 (2.9%) |  |
| 4 | 4 (5.3%) | 0 (0%) |  |
| 5 | 5 (6.6%) | 2 (5.9%) |  |
| 6 | 9 (11.8%) | 7 (20.6%) |  |
| 7 | 1 (1.3%) | 1 (2.9%) |  |
| Missing | 57 (75.0%) | 23 (67.6%) |  |
| WHO-OSCI D14 |  |  | 0.055 |
| 5 | 3 (3.9%) | 0 (0%) |  |
| 6 | 1 (1.3%) | 4 (11.8%) |  |
| 7 | 2 (2.6%) | 1 (2.9%) |  |
| Missing | 67 (88.2%) | 29 (85.3%) |  |
| WHO-OSCI D21 |  |  | 0.487 |
| 4 | 1 (1.3%) | 0 (0%) |  |
| 5 | 0 (0%) | 0 (0%) |  |
| 6 | 2 (2.6%) | 3 (8.8%) |  |
| 7 | 2 (2.6%) | 1 (2.9%) |  |
| Missing | 71 (93.4%) | 30 (88.2%) |  |
| WHO-OSCI D28 |  |  | 0.525 |
| 4 | 0 (0%) | 1 (2.9%) |  |
| 5 | 1 (1.3%) | 0 (0%) |  |
| 6 | 1 (1.3%) | 1 (2.9%) |  |
| 7 | 1 (1.3%) | 2 (5.9%) |  |
| Missing | 73 (96.1%) | 30 (88.2%) |  |
| WHO-OSCI DISCHARGE |  |  | 0.213 |
| 1 | 50 (65.8%) | 18 (52.9%) |  |
| 2 | 19 (25.0%) | 8 (23.5%) |  |
| 4 | 1 (1.3%) | 2 (5.9%) |  |
| 8 | 6 (7.9%) | 6 (17.6%) |  |
